# A review of climate change effects on practices for mitigating water quality impacts

**DOI:** 10.2166/wcc.2022.363

**Published:** 2022-03-22

**Authors:** Thomas Johnson, Jonathan Butcher, Stephanie Santell, Sara Schwartz, Susan Julius, Stephen LeDuc

**Affiliations:** a EPA ORD, 1200 Pennsylvania Ave NW, Washington, DC 20460, USA; b Tetra Tech, Inc., P.O. Box 14409, Research Triangle Park, NC 27709, USA; c EPA OW, 1200 Pennsylvania Ave NW, Washington, DC 20460, USA; d EPA ORD, 109 TW Alexander Dr., Research Triangle Park, NC 27709, USA

**Keywords:** adaptation, best management practices, climate change, conservation practices, resilience

## Abstract

Water quality practices are commonly implemented to reduce human impacts on land and water resources. In series or parallel in a landscape, systems of practices can reduce local and downstream pollution delivery. Many practices function via physical, chemical, and biological processes that are dependent on weather and climate. Climate change will alter the function of many such systems, though effects will vary in different hydroclimatic and watershed settings. Reducing the risk of impacts will require risk-based, adaptive planning. Here, we review the literature addressing climate change effects on practices commonly used to mitigate the water quality impacts of urban stormwater, agriculture, and forestry. Information from the general literature review is used to make qualitative inferences about the resilience of different types of practices. We discuss resilience in the context of two factors: the sensitivity of practice function to changes in climatic drivers, and the adaptability, or relative ease with which a practice can be modified as change occurs. While only a first step in addressing a complex topic, our aim is to help communities incorporate consideration of resilience to climate change as an additional factor in decisions about water quality practices to meet long-term goals.

## 1. INTRODUCTION

An increased frequency of extreme precipitation events has been observed in North America and event attribution suggests that the risk is increasing due to climate change (Kirschmeier-Young & Zhang 2020; van Oldenborgh *et al*. 2021). While headlines focus on large-scale disasters such as floods and droughts, subtler impacts of changing precipitation patterns on water and water quality are also important. A changing climate will have direct and cascading effects on water quality in the United States (Kaushal et al. 2010; Isaak *et al*. 2012; Coffey *et al*. 2019; Paul *et al*. 2019). Assessing potential risks, however, is complicated as water quality is influenced by multiple interacting climatic, watershed (e.g., physiographic setting, land use), and human factors (Melillo *et al*. 2014; USGCRP 2018) with high spatial and temporal variability.

To protect and restore aquatic systems, significant investments are made to conserve natural resources and to abate pollutant losses from the landscape. In an urban setting, managers use different Best Management Practices (BMPs) to reduce and/or filter pollutants from stormwater runoff. In agricultural areas, multiple practices are generally used together in conservation practice systems to conserve natural resources and to abate pollutant losses from the landscape. In forested areas, BMPs typically focus on reducing runoff and pollutant loads associated with silvicultural activities. Hereafter, we use the general term ‘water quality practice’ to refer to each.

In any landscape, water quality practices can be implemented independently, in parallel or in series to mitigate existing or prospective impacts of land use and other human impacts on water quality. Practices are typically designed based on existing problems (e.g., flooding or water quality impairment) or in anticipation of potential problems based on experience with similar sites (e.g., expected impacts from new urban development). These decisions often assume water quality practices and conservation systems will function as observed under historical climatic and hydrological conditions.

The effects of climate change on water quality will vary in different regional and watershed settings (Johnson *et al*. 2015; Coffey *et al*. 2019), and are strongly mediated by physical setting (e.g., geology, topography), land use, and the human use and management of water. Effects on water quality are also better understood for some attributes than others; e.g., water temperatures are likely to increase in much of the US, whereas changes in nonpoint loading of nutrients, sediment, and other pollutants from upland sources to waterbodies will vary across locations and are closely linked to changes in precipitation. Climate change can also directly affect water quality practices and systems. In many locations, practices designed for historical climatic conditions may not have the capacity to handle increases in heavy precipitation or otherwise function as intended.

Managing the risk of future impacts will require anticipating and planning in advance for adaptation. The complexity and inherent uncertainty of the problem, however, is a challenge to decision makers seeking actionable information. Global climate models (GCMs) are simulated representations of the climate system, and while there is substantial and increasing skill at projecting future conditions, projections are subject to uncertainty (Melillo *et al*. 2014). As a result, water managers need to make decisions about practices in the context of uncertain future climatic conditions. Flexible, risk-based approaches that consider a range of potential future climatic and hydrological conditions are required. Central to this is a need to identify resilient, practice-based strategies that will perform as intended over time across a range of climatic conditions. In this context, resilient practices are those that can tolerate greater disturbance before function is compromised or have a greater capacity to recover from or be adapted to restore diminished function. Practices that can be easily modified or expanded over time in a phased or modular way also add resilience.

Many water quality practices function via combinations of physical retention, filtration, plant growth, biological uptake, and other mechanisms that are sensitive to changes in precipitation and temperature. Accordingly, climate change effects on different types of practices will vary depending on the processes involved, e.g., depending on whether they function by ‘treatment control’ or pollutant ‘source control’. Treatment control practices are likely to be affected by changes in pollutant removal efficiency (here defined as the ratio of pollutant mass leaving compared to entering a practice). Pollutant removal efficiency may change as a result of altered amounts and timing of runoff and pollutant loads (e.g., Walsh *et al*. 2014), and changes in the physical and/or biological functioning of practice load reduction mechanisms. Practice performance is also known to vary due to climatic variability (e.g., daily, seasonal, interannual, and decadal oscillations affecting weather). Additionally, some practices with a focus on source control (e.g., erosion control) are likely to be affected by climate change, including changes in the location of pollutant critical source areas, while others may not be (Arisz & Burrell 2006; Garbrecht *et al*. 2014). The literature is limited on in-depth examinations of the interactions of hydrology, plant growth, and pollutant removal under future climate; most available studies include observations of practice sensitivity to climatic variability, largely through hydrologic/water quality model simulations driven by future climate scenarios that do not necessarily predict future performance.

In this review, we summarize the literature on how climate change could affect water quality practices commonly used to control and/or treat runoff and pollutant loading from urban stormwater, agriculture, and forestry. Information from the literature review is used to make qualitative inferences about the resilience of different types of practices. While significant gaps in understanding remain on this topic, periodic review and synthesis can be useful to help inform management decision making. Results are intended to help communities and resource managers better incorporate consideration of climate resilience when selecting practices to meet their water quality goals, and to identify areas in need of further research. This paper contributes to a growing understanding of potential future hydroclimatic changes on water quality and the effectiveness of management responses for addressing water quality impairment.

## 2. REVIEW OF CLIMATE CHANGE EFFECTS ON PRACTICES

Our analysis focused on peer-reviewed research from scientific journals and official government agency reports. Google Scholar, Microsoft Academic, Scopus, and AGRICOLA were used to identify candidate documents. Keyword searches were conducted with general terms including ‘climate change’, ‘BMPs’ or ‘best management practices’, and searches on individual practice types. The titles and abstracts of candidate documents were then screened for relevance based on their applicability to study questions regarding climate change effects on practice performance. Note that we address only structural practices that are designed to limit runoff and associated pollutant loading from upland areas. We do not address instream practices such as stream restoration, although these can be important components of an overall management strategy.

Search results reveal relatively few studies that directly examine the effects of future climate change on practice performance. However, there is substantial indirect information on practice sensitivity to observed weather and climate variability, including changes in temperature, hydrology, and other climate-relevant factors. These types of studies were taken as proxies for sensitivity to future climate change, even where climate change is not explicitly addressed (e.g., the review of Wang *et al*. (2019) on bioretention design for pollutant removal efficiency). The following sections summarize the literature identified for urban, agricultural, and forestry settings.

### 2.1. Urban BMPs

The impacts of climate change on urban stormwater infrastructure (including both BMPs and the broader stormwater conveyance system) depend on variations in the magnitude and frequency of peak flows, which may result from changes in rainfall intensity or from alterations to the snowmelt regime (e.g., Hamlet & Lettenmaier 2007). Other determinants include site use, the amount and location of impervious surfaces, soil type, vegetative cover, and slope.

BMPs implemented to reduce the adverse impacts of urban stormwater can be loosely grouped as ‘gray’ or ‘green’ infrastructure. Traditional gray stormwater infrastructure uses single-purpose, hard structures including detention basins and storm sewers to convey runoff away from the built environment. Gray infrastructure can be effective, particularly for large events, but is also highly sensitivity to design specifications (e.g., storage volume), and difficult to modify in the event of changing conditions. Conversely, nature-based or green infrastructure uses vegetation and soil to reduce and treat stormwater runoff at its source (e.g., bioretention). Green infrastructure is typically more decentralized and can be implemented at local to larger, neighborhood spatial scales. Green infrastructure is particularly effective, in tandem with gray infrastructure, for managing smaller events while delivering other environmental, social, and economic co-benefits. In some cases, BMPs such as wet ponds combine both gray (detention) and green (biological) processes (e.g., in ponds with a wetland fringe).

Climate change is expected to result in a more vigorous hydrologic cycle, including increases in the intensity and frequency of heavy precipitation events (USGCRP 2017; Hayhoe *et al*. 2018). For gray stormwater management practices such as detention ponds, the primary focus in the literature has been on potential changes in storm magnitude and frequency. The design of urban stormwater BMPs typically begins with consideration of rainfall recurrence intervals, which may be translated into design storm specifications or runoff depth (e.g., Claytor & Schueler 1996; Kadlec & Knight 1996; Berndtsson 2010; Gallo *et al*. 2012; Hunt *et al*. 2012). Practices are designed to achieve a level of service or performance associated with controlling a design storm, combination of design storms, and/or runoff depth to reduce flooding, stream erosion, and pollutant loading. If the rainfall recurrence relationships change, the design standards must change as well to preserve retention and treatment contact times. While many studies focus on the potential effects of changes in precipitation intensity and duration (e.g., Huard *et al*. 2010; Arnbjerg-Nielsen 2012; Srivastav *et al*. 2014), it is preferable to evaluate infrastructure needs based on joint changes in precipitation, evapotranspiration (ET), and antecedent soil moisture (Forsee & Ahmad 2011).

Arisz & Burrell (2006) summarize the basic facets of urban stormwater infrastructure planning and design under climate change. They first distinguish between major and minor drainage systems, where the minor drainage system operates at the local scale and is designed to convey runoff from more frequent, less severe (<10-year recurrence) storm events, and the major drainage system is designed to convey stormwater runoff from less frequent, larger storms when the capacity of the minor storm drainage system is exceeded. The minor drainage system includes both gray and green infrastructure, and protects uses of streets, parking lots, and other developed areas under normal circumstances. The major drainage system (mostly open channels, rivers and streams, and regional-scale detention ponds) protects against catastrophic losses when the minor system is surcharged during larger storm events.

It is often thought that the major drainage system requires the largest capital costs and longest planning horizon, and therefore may be most susceptible to climate change. However, Arisz & Burrell (2006) contend that this is not the case, other than leaving enough room available for future upgrades. In contrast, the hard or gray portions of the minor drainage can be very difficult to redesign or expand because they are intimately entwined with other utilities and site layouts, and therefore may actually require more thought and planning at the development or redevelopment stage to allow for climate adaptation.

One of the advantages of green infrastructure is greater flexibility on sizing for future climate, including use of modular design that allows additional distributed components to be added to (or subtracted from) an overall BMP plan to adapt to changing conditions. Modular designs allow for adaptive management of systems and can reduce the long-term costs and other constraints of losing available treatment areas. However, as with gray infrastructure, the performance and life cycle of green infrastructure BMPs can potentially be altered by climate change. Many studies show the benefits of distributed green infrastructure for mitigating projected increases in storm intensity and runoff (Gill *et al*. 2007; Semadeni-Davies *et al*. 2008a, 2008b; Forsee & Ahmad 2011; Pyke *et al*. 2011; Semadeni-Davies 2012; Newcomer *et al*. 2014; Rossman 2014; USEPA 2018). However, few studies are available that have examined in detail the effects of climate on the biological processes that contribute to urban BMP performance in plant- and soil-based systems such as bioretention cells.

Limited direct information is available in the literature about the types of urban BMPs that are most sensitive to changes in precipitation, temperature, and other climatic drivers, although several studies have begun to investigate this issue. Sohn *et al*. (2019) conducted a systematic review and meta-analysis of literature (through 2017) on the influence of climate on the effectiveness of green infrastructure in controlling runoff volume and peak flow. The results suggest that changing storm frequency will have a greater effect on runoff volume than on peak discharge rates, while the capacity of green infrastructure to reduce both volume and peak discharge is expected to decrease with greater storm intensity and frequency due to increased periods of soil saturation or antecedent moisture content. Storm duration was also identified as an important factor. In general, changes in climate that increase antecedent soil moisture will reduce the capacity of green infrastructure BMPs to control runoff volume and peak flow for smaller storms, but have little effect on peak flows for larger storm events.

Semadeni-Davies (2012) studied wet ponds and bioretention cells, concluding that oversizing a wet pond to accommodate the full range of projected future climate would be cost prohibitive, but bioretention cells may prove more flexible. Tirpac *et al*. (2021) evaluated the potential performance of bioretention cells under future climate conditions in Knoxville, TN, and found that substantial increases in bioretention surface area would be needed to maintain current levels of service (as measured by infiltration and surface overflow volumes). A modeling study (USEPA 2018; Job *et al*. 2020) provided insights into the potential impacts of changes in precipitation events on performance stormwater BMP systems. Different types of urban stormwater BMPs were modeled under current and mid-21st century precipitation scenarios, allowing comparison of the responses of conventional gray and green practices. In many settings, a combination of gray and green infrastructure practices was the best approach for meeting multiple objectives for water quality and channel protection at the least cost. Consistent with this finding, many communities with large, combined sewer systems have used a combination of gray infrastructure-based storage and green infrastructure practices to reduce sewer overflows.

Job *et al*. (2020) also showed that faced with increased rainfall and stormwater runoff volume, conventional gray and green infrastructure BMPs generally removed greater runoff volumes and pollutant mass compared to current conditions. However, overall site export rates of runoff volume and pollutant mass still increased (i.e., the BMP did not remove 100% of the additional runoff/pollutant load resulting from increased precipitation) despite improved volume/mass removal. This suggests that in areas exposed to increased precipitation, existing stormwater infrastructure may need to be expanded to account for increases in runoff and pollutant loading. For new development, initial site design may need to be configured to allow addition or expansion of existing stormwater treatment BMPs if the need arises in the future.

Sarkar *et al*. (2018) applied a biogeochemical simulation model, known as the Regional Hydro-Ecologic Simulation System, or RHESSys (Tague & Band 2004), to assess green infrastructure performance under a range of mid-century climate change scenarios. Results showed that green infrastructure designed for current climate will reduce surface runoff and increase water loss to the atmosphere as evapotranspiration under future conditions. For scenarios where precipitation and runoff increased, simulations suggest that green infrastructure provides substantial mitigation of increases in pollutant loading associated with climate change. However, green infrastructure under future climate scenarios did not achieve the levels of protection expected for these practices under current climatic conditions. RHESSys simulations in this study also demonstrated that green infrastructure can provide advantages relative to gray, hard structures for managing stormwater due to greater flexibility and shorter design time horizons. This provides greater opportunity for expanding or modifying practices over time as climate changes. Green infrastructure can also provide co-benefits, such as mitigation of urban heat island effects, preservation of habitat continuity, and provision of recreational amenities that can increase the benefit-to-cost ratio relative to gray practices.

### 2.2. Agricultural conservation practices

Agricultural conservation practices are typically designed to manage multiple outcomes, including controlling pollutant loads from agricultural production while improving crop growth and soil health (USDA-NRCS 2015). Conservation practices for controlling runoff and pollutant loads in agricultural landscapes include pollutant source avoidance, reduction, and treatment and/or trapping practices. Structural practices trap and may treat pollutants (e.g., constructed wetlands) and nonstructural practices avoid or control the movement of pollutants (e.g., nutrient management, conservation tillage). Many agricultural practices also control or reduce pollutant exports through the manipulation of soil health, soil moisture, and plant management.

Climate change effects on the performance of conservation practices include increased erosive flows that reduce vegetative ground cover used to filter runoff. The timing of runoff events relative to seasonal agricultural operations such as tillage, fertilization and harvest also have a substantial impact on the effectiveness of certain practices, together with changes in the overall soil moisture balance that affect the need to drain or irrigate croplands to maintain productivity. Hatfield & Prueger (2004) evaluated current soil management practices in the corn belt (crop residue, no-till, incorporation of manure) and concluded that their effectiveness would decrease under future climatic conditions. Nearing *et al*. (2004, 2005) and O’Neal *et al*. (2005) also projected future increases in soil erosion in mid-western US states and stressed the importance of soil cover, which itself may be affected by climate, in mitigating soil loss. Rounsevell *et al*. (1999) stressed the importance of good land management practices to combat the impacts of climate change on soils and agriculture. Maintaining and/or improving soil health and natural hydrologic functions is key to protecting or improving water quality in agricultural landscapes. Soil amendments’ impact on water quality are also being examined in many areas as they support increased infiltration rates, overall soil health and reduced hazard mitigation (flooding).

Garbrecht *et al*. (2014) and Tomer *et al*. (2014) concluded based on reviews of studies conducted by the U.S. Department of Agriculture (USDA) Conservation Effects Assessment Project (CEAP) that anticipated increases in rainfall amounts and intensity associated with climate change will stress many conservation practices. In a two-watershed monitoring study, Mellander *et al*. (2015) found that rainfall-runoff partitioning was the most important factor influencing phosphorus loss. The authors suggested that surface versus subsurface flow pathways, which may change with climate, are more important than other factors known to influence phosphorus loss such as soil phosphorus content. In each case, studies advocate for wider adoption of technologies that minimize soil disturbance (Tomer *et al*. 2014), and increased use of practices that promote protective ground cover such as conservation tillage, including no-till operations, and strip cropping to mitigate climate change effects on agricultural sediment and pollutant loads. Simulations by Yasarer *et al*. (2017) also suggest climate change will generally increase sediment and nutrient loads, and that cover crops, double cropping, and no-tillage practices can mitigate these impacts. Both Tomer *et al*. (2014) and Garbrecht *et al*. (2014) stressed the need for additional studies to better understand the interactions among land use, water quality, conservation practices, and climate change.

Modeling studies incorporating climate change scenarios provide additional insights into potential changes in the functioning of conservation practices, including effects on the performance of specific practices in reducing pollutant loading to waterbodies (e.g., Soil and Water Assessment Tool, SWAT; Neitsch *et al*. 2011). It must be noted, however, that simulated changes in practice performance are conditional on the ability of the model framework to correctly represent flow and pollutant removal processes in conservation practices and the effects of changes in water balance, temperature, and biological processes. Modeling studies provide a useful benchmark, but when possible results should be weighed against all available sources of information.

Liu *et al*. (2016) reviewed modeling studies on the effects of climate change on the hydrologic performance of conservation practices. The study suggests that, due to changes in hydrologic regime, practice preference may shift from practices that focus on dissolved pollutants to those that focus on reducing soil erosion, sediment yield and the transport of sediment-bound pollutants. Extreme flooding may render some practices ineffective; conversely, increased drought may render others ineffective at reducing nonpoint source pollution if streams dry up. Changes in climate could also shift some conservation practices from functioning as sinks to sources of pollutants if they are destroyed by flooding, or if accumulated pollutants flush out during extreme events. These changes also have implications for the siting of practices in addition to selection or modifications. The study also noted that the relative cost-effectiveness of different types of practices could change under future climate conditions, while also recognizing the potential uncertainty in modeling studies.

Other modeling studies have evaluated how practice performance may be affected by climate change in different agricultural settings. In Tuttle Creek Lake watershed in Kansas/Nebraska, simulations suggest despite climate-driven increases in streamflow, sediment, and nutrient loads, practices will continue to provide reductions with little change in removal efficiencies (Woznicki *et al*. 2011; Woznicki & Nejadhashemi 2012, 2014). Similar results were shown for Shell and Logan Creek watersheds in Nebraska (Van Liew *et al*. 2012). Bosch *et al*. (2014) simulated practice responses to climate change scenarios using the SWAT model in four rivers draining to Lake Erie (the Raisin, Maumee, Sandusky, and Grand Rivers). SWAT simulations suggested that conservation practices, such as no-till practices, cover crops, and filter strips will become less effective in removing pollutants under future climate due to increased flows, lessened contact time, and reductions in filter strip vegetation density under drought. In contrast, in the Chesapeake Bay watershed, Lee *et al*. (2017) predicted increases in nitrate load reduction by winter cover crops. Increases were attributed to increases in plant biomass in response to warmer winters and increased atmospheric CO_2_ concentrations. In the U.S. Midwest, SWAT modeling studies by Wallace *et al*. (2017) and Chiang *et al*. (2012) suggested that practice performance for total nitrogen and total phosphorus reduction will be generally stable under future climatic conditions, but sediment removal efficiency may decline. Simulations by Dakhlalla & Parajuli (2016) showed that practice effectiveness in reducing peak flows may decline significantly with an increase in precipitation or CO_2_ concentration, while increased temperature or reduced precipitation did not have significant impacts on practice performance.

Schmidt *et al*. (2019) used SWAT simulations to investigate the effects of projected mid-century and late-century climate change on conservation practices in two small, agricultural watersheds in the Minnesota Corn Belt and Georgia Coastal Plain. The study considered conservation tillage, no-till, vegetated filter strips, grassed waterways, nutrient management, winter cover crops, and drainage water management practices. Results suggest that anticipated increases in air temperatures and changes in seasonal precipitation may exacerbate agricultural pollutant loads, resulting in higher sediment and nutrient loads. Removal efficiencies for many conservation practices decline due to more intense precipitation and runoff events, leading to increased sheet and rill erosion, increased dissolved constituent transport, and changes in plant growth due to altered soil moisture and temperature. For example, sediment removal efficiencies decreased by 1.4–6.1% for climate scenarios when averaged across all practice types studied by Schmidt *et al*.; however, performance did not degrade for all practice types. Winter cover crops in Minnesota were predicted to be healthier under a warmer future climate, which improved sediment removal efficiency by 3% by the late 21st century.

Coffey *et al*. (2020) extend the analysis of conservation practice performance to microbial pathogens. Changes in filtration efficiency for pathogens are expected to be similar to those for sediment, but the analysis is further complicated by the effects of temperature on pathogen survival and die-off. At the same time, reductions in recreational season flows may decrease the dilution of direct pathogen inputs.

### 2.3. Forestry BMPs

Undisturbed forest land is generally associated with good water quality (Hewlett 1982). Forests reduce storm runoff due to high rates of canopy interception (of precipitation) coupled with higher rates of infiltration associated with soil macropores. Soil erosion is also limited by the stabilizing effects of roots and resistance to soil detachment provided by the tree canopy and surface litter. However, human activities associated with silviculture, such as timber harvesting and road building, can expose and compact soil, resulting in increased rates of direct runoff, erosion, and export of other pollutants (Elliot *et al*. 2009).

There is a relatively large amount of literature addressing climate change effects on forest ecosystems; however, few authors have focused on how the performance of BMPs in forested lands may be impacted by climate change. Potential effects include physical/engineering and ecosystem-based performance components. Findings regarding several important classes of forestry BMPs are summarized below.

#### Streamside Management Zones (SMZs):

SMZs reduce sediment and pollutant loading to streams during and after forest harvest operations. They also provide shade reducing heat input to streams. Forest harvest disturbance in SMZs can cause increased thermal input and elevated water temperatures. Harvest disturbance can also alter hydrology and increase sediment and nutrient loads to streams. Riparian buffer requirements are designed to mitigate these impacts; however, future climate change could reduce the efficacy of SMZs and other forest management practices in protecting water quality and aquatic ecosystems.

SMZs will likely be impacted by variable environmental conditions caused by climate change in both direct and indirect ways. Stream buffers are less effective at pollutant removal where flow concentration/convergence or velocity increase (Liu *et al*. 2008). Buffers become less effective with frequent rainfall because their capacity to absorb and filter runoff is lower when the soil is already saturated (Liu *et al*. 2008). Major precipitation events may also form gullies in SMZs which disrupt the shallow sheet flow needed for optimal sediment trapping and nutrient removal (Osborne & Kovacic 1993). Indirect effects on SMZs include climate-based influences on risk of fires, disease, and pests that may result in change in vegetation density, type (e.g., species), and health in SMZs (Breshears *et al*. 2005). Short-term impacts of events such as catastrophic fires, the risk of which is itself increased by a warming climate, may render SMZs ineffective until regrowth occurs.

Butcher *et al*. (2016) combined climate projections with a stream water temperature model (QUAL2Kw) and SMZ scenarios to evaluate future water temperature conditions in the South Fork Nooksack River in northwest Washington state. Model simulations showed that restoration of 100-year potential riparian shade zone would mitigate warming effects in the short term (e.g., next 20 years) but would not keep pace with the projected increases in air temperature over the next century. Yonce *et al*. (2020) used a combined modeling approach based on an upland watershed model (SWAT), a riparian vegetation shading model, and an instream response model (QUAL2Kw) to evaluate the performance of SMZs under future climate conditions for a forested watershed (Lookout Creek) in western Oregon. Absent changes to SMZs, climate change alone increased sediment and nutrient loading rates from 7 to 10% by the end of the 21st century. With existing SMZ conditions, future maximum water temperatures increased from 17 to 38% during critical hot, dry summer periods.

Although it is difficult to predict how SMZs will respond to climate change, buffers can be adapted to better withstand effects of climate change. Maintaining or improving riparian vegetation health in SMZs is important to sustain water quality benefits. Establishing native species and ensuring genetic diversity in the composition of buffers through horticultural techniques may increase the functionality and resilience of buffers (Seavy *et al*. 2009). Altering the composition of buffers to contain a spectrum of species with a range of hydrologic, temperature, and other tolerances may also increase resilience to climate change (Seavy *et al*. 2009). Overall, however, SMZs have qualitatively high susceptibility to climate change with limited adaptability options.

#### Wildland Fire Management:

While demand for fire suppression and fuel management may increase with climate change, ability to control fire may be constrained. For example, increased frequency and prevalence of drought may limit the applicability of thinning and controlled burns (Swanston *et al*. 2016). Winds may also increase in intensity and unpredictability, making it more difficult to conduct controlled burns (Bessie & Johnson 1995). Additionally, the prevalence of invasive species and pest infestations may further increase the difficulty of administering controlled burns and thinning (Ferrell 1996).

As drought and forest fires are expected to increase in frequency and severity, the scale of fuel management may need to shift from small scale at the stand level to large scale at the landscape level (Black 2004). However, there is a lack of agreement on standards for large-scale thinning and other fuel management activities, suggesting that more research should be conducted to ensure that these practices will be beneficial in response to climate change effects (Doppelt *et al*. 2009).

#### Timber Harvesting:

Climate change is expected to have varied impacts on forest water balance, leading to uncertainty in how harvest BMPs will be affected. Vose *et al*. (2012) performed a long-term hydroclimatic change assessment of interactions among climate, streamflow, and forest management. The study found that clearcutting may exacerbate drought impacts to streamflow in high elevation forested areas, but not in low elevation areas. Neary *et al*. (2009) compared streamflow between undisturbed and harvested sites across a range of precipitation regimes and found an approximately 20% average increase in streamflow for harvested sites in wetter climates over the first year, although long-term effects of clearcutting on soils may result in streamflow below pre-harvest levels (Jones & Post 2004).

SFC (2010) presents general harvesting guidelines that consider the effects of climate change on forests, with the following strategies: harvesting should be conducted at a smaller scale than at present, natural regeneration should be preferred, harvesting operations should be designed to mitigate potential disturbances, regular harvesting activities should be maintained, and rotation periods should be increased in coppices. The type of harvesting recommended (i.e., clearcutting, whole tree, etc.) will depend largely on-site conditions (SFC 2010).

#### Post-Harvest Site Renewal:

Forests are often allowed to regenerate naturally following disturbances such as harvesting if enough seed trees are present; in other cases (e.g., after clear cuts), stand regeneration may include active planting. Success of forest regeneration is likely to be impacted by climate changes, such as increased frequency and intensity of floods and drought (USGS 1997; Ogden & Innes 2008). More frequent storms and drought conditions would harm canopy trees and reduce the ability of seedlings to establish (Swetnam & Betancourt 1997; USGS 1997; Borja 2014). Seedlings will also likely have to compete with more vines and, potentially, invasive species as climate change continues (Rustad *et al*. 2011).These factors make it uncertain that forests will regenerate with the same structure as before they were harvested (Rustad *et al*. 2011).

There are various recommendations for natural regeneration practices to help adapt to climate change. One option is to assist plant migration as habitats become too warm or dry (UAF 2017). Other options are to increase the length of rotation period, select more appropriate or hardy tree species, control weeds, increase genetic diversity, maintain the original population size and reproductive potential, and avoid effects of drought by planting in the fall season and placing saplings in pots (SFC 2010). An alternative for commercial forestry is to practice short rotation clear cuts followed by active planting to speed regrowth. Planting can enable selection of cultivars with superior tolerance to anticipated changes in climate.

#### Forest Road BMPs:

Dirt and gravel roads can be a major source of sediment loading in forest areas. Decisions about forest road maintenance and closure are affected by environmental conditions such as patterns of precipitation and runoff, terrain instability, and forest growth and productivity (Ogden & Innes 2008). Increases in heavy precipitation will likely make road BMPs less effective; roads and their drainage systems are not designed for increased precipitation and erosion (Mote *et al*. 2003). This would likely lead to the increased risk of landslides from roads (Dale *et al*. 2001). Moreover, improvements aiming to reduce the velocity of runoff would likely become less effective in cases of extreme precipitation (Grace & Clinton 2007). Additionally, maintenance and removal of roads are invasive procedures that may no longer be considered acceptable in areas where climate change has made native species more sensitive to disturbance (Grace & Clinton 2007). Changes resulting in inadequate culvert design can also result in increased erosion.

The main way to adapt road BMPs to climate change is to stay ahead of maintenance needs (Spittlehouse & Stewart 2003). Environmental factors are now considered more often when forest roads are designed, and various models have been developed to help balance multiple factors during design (Akay & Sessions 2005; Grace & Clinton 2007; Gumus *et al*. 2008). Road maintenance decisions would be improved by considering increases in precipitation and runoff effects as well as impacts of changes in freeze/thaw conditions (Spittlehouse & Stewart 2003).

## 3. RESILIENCE OF WATER QUALITY PRACTICES

The literature review in the previous section suggests a variety of threats to the performance of water quality practices under future climate conditions. Faced with a real but inherently uncertain risk of impacts, there is growing interest in strategies for increasing the resilience of water quality practices to a range of future climatic conditions and events.

Practice resilience can be defined in different ways depending on goals. In this section, as a heuristic framework, we define and discuss practice resilience in the context of two criteria: (1) the sensitivity of practice pollutant reduction performance to changes in climatic drivers and (2) the adaptability, or relative ease and time required to modify or redesign a practice (e.g., planning horizon, project lifespan). Application of this framework, while qualitative, allows a simple, screening level assessment of practice resilience to support water quality management decisions.

The first criterion, sensitivity to change, identifies the degree to which management practices will be sensitive to alterations in air temperature, precipitation, and other climatic drivers. The relative sensitivity of different practices is estimated based on a qualitative, conceptual understanding of the sensitivity of key, underlying physical/engineering and ecosystem processes affecting pollutant removal. For example, gray stormwater management practices, such as stormwater detention ponds, capture a proportion of urban runoff, and control runoff volume and pollutant loads over time by some combination of settling, infiltration, natural decay, and evaporation. Increases in precipitation can result in increased runoff, decreased practice retention and treatment times, and insufficient practice storage capacity. Green infrastructure practices in most locations are used in conjunction with gray infrastructure to achieve effective volume control. Most green infrastructure-based practices, although not designed for managing large volumes of water, also include a storage component in addition to ecosystem processes (e.g., plant uptake and transpiration) to control and treat runoff volumes and pollutant loads. Increases in precipitation can have similar effects on green infrastructure practices, reducing practice retention and treatment times.

Key ecosystem-based factors/processes that can affect practice function are the water cycle, mineral/nutrient cycle, community dynamics (i.e., functional biodiversity, mostly associated with soil biology), and energy flow (i.e., conversion of solar energy to biomass). If any one of these processes are in decline, either through poor management practices or changing climatic conditions, the system is more likely to experience impaired performance results via increased runoff, sediment loss, or nutrient leaching. For each type of practice assessed, potential effects of changes in climatic variables on key ecosystem processes were examined by considering effects on soil ground cover, soil organic matter, and the humidity distribution and brittleness scale (Savory & Butterfield 1999). Soil organic matter is influenced by changes in ambient temperature, humidity, and the frequency/magnitude of extreme rainfall events, which can influence the performance of agricultural conservation systems. The effects of increasing brittleness on ecosystems, while complex, were assumed to include reduced soil cover and biological activity leading to decreases in soil organic matter, impacts on soil surface microclimate and potential increases in nutrient leaching.

The second criterion, adaptability, identifies practices that can be more readily modified or redesigned. More specifically, adaptability refers to the complexity, cost, and time required to modify or redesign a practice. Adaptable practices allow decision makers to remain flexible and act only as necessary over time as new information becomes available, thus reducing the risk of over-investment in practices based on a worst-case scenario that may never occur. In many locations certain hard, long-lived, or otherwise relatively inflexible practices (e.g., engineered storage structures) are critical infrastructure necessary to meeting water quality goals. In combination with hard infrastructure, however, more adaptable practices provide an opportunity to hedge against future climate-related risk, and are a key component of flexible, modular practice design plans for adaptive risk management. Factors affecting practice adaptability include practice design type (e.g., certain hard, structural features are less adaptable than nonstructural features), service lifespan (e.g., long lifespan is generally less adaptable than a short lifespan), and the complexity of ecosystem processes that influence treatment functions (e.g., complex systems can exhibit greater resilience if the complexity provides redundancy in functional ecosystem processes; selection of vegetation better adapted to changing conditions has the highest potential performance and resilience to a wider range of climatic conditions).

Few studies explicitly assess the sensitivity or adaptability of different types of water quality practices to climate change. In most cases, however, certain qualitative inferences can be drawn based on mechanistic knowledge of key functional processes (e.g., physical retention, filtration, biological uptake) and projected climate change. The following sections summarize the sensitivity and adaptability of practices in urban, agricultural, and forestry settings.

### 3.1. Urban BMPs

Urban BMPs range from engineered storage structures to green infrastructure that is based primarily on soil and vegetative components. Regardless of the type, most urban BMPs are limited by space and site constraints and, either alone or in combination with other management measures, will need to maintain a certain level of performance to control peak flow volumes generated by impervious surfaces. Thus, most urban BMPs will have sensitivity to physical process factors such as changes in storm volume that limit retention volume or limit contact time for settling and filtration, while green infrastructure that depends on plant growth and soil health for its function will also be sensitive to changes in ecosystem processes.

Key considerations relevant for urban stormwater management as reported in the National Climate Assessments (Melillo *et al*. 2014; USGCRP 2017) include:

- Average *surface air temperatures* will increase considerably, although the magnitude of change varies widely among future emissions scenarios in specific areas.
- The recent trends of *increased occurrences of extreme weather events* such as heat waves, droughts, rainfall intensity, hurricanes, flooding, and heavy snowfall are likely to continue.
- The *location, timing, and amounts of precipitation* will also change as temperatures rise. There is considerable uncertainty in model forecasts of trends in precipitation, but, in general, wet regions are projected to become wetter while dry regions are projected to become drier. The northern part of the US is projected to see more winter and spring precipitation, while the southwestern US is projected to experience less precipitation in the spring. Reduced summer precipitation is projected for parts of the US, including the Northwest and southern Great Plains.
- Increased temperatures and changing precipitation patterns will alter *soil moisture*, which is important for the performance of some green infrastructure practices. Areas that experience reduced precipitation coupled with increased evapotranspiration may experience significant deficits of soil moisture. Areas with more frequent and intense storms will exhibit periods with higher soil moisture and saturated conditions which may affect vegetation selection, growth, and pollutant removal capacity.

Table 1 gives a summary of factors affecting the resilience of selected urban BMPs. Additionally, more detailed information about the sensitivity, adaptability, and potential adaptive responses for different types of urban practices, including reference citations, is available in the Supplementary material.

#### 3.1.1. Sensitivity

The sensitivity of urban stormwater infrastructure (including both BMPs and the broader stormwater conveyance system) to climate change depends first on changes to magnitude and frequency of peak flows, which may result from changes in rainfall intensity and time between events or from alterations to the snowmelt regime (e.g., Hamlet & Lettenmaier 2007). Other determinants in urban stormwater infrastructure design include site use, the amount and location of impervious surfaces, soil type, vegetative cover, and slope.

Increased impervious surfaces and enhanced connectivity of drainage networks lead to higher stormwater runoff volume and peaks, and enhanced pollutant loads (e.g., Walsh *et al*. 2005). Different types of BMPs can be implemented to reduce the adverse impacts of urban stormwater. Traditional gray stormwater management infrastructure uses single-purpose, hard structures to treat and convey runoff. In general, gray stormwater management practices, such as stormwater detention ponds, address both flow volume and quality by capturing a proportion of stormwater runoff and holding it for enough time to allow control of release flows and pollutant loads by some combination of settling, infiltration, natural decay, and evaporation. These engineered solutions can be effective but difficult to modify to meet changing conditions.

Conversely, green approaches use vegetation, soil, and other media to manage rainwater and pollutant runoff near its source. Green approaches to urban stormwater management typically combine some amount of physical storage with ecosystem processes (e.g., plant uptake) that help to control and treat runoff volumes and pollutant loads. Green infrastructure can also provide co-benefits (e.g., mitigation of urban heating, carbon and nitrogen sequestration, and natural habitat), cost savings, and flexibility as compared to engineered, hard structures. A review of 17 case studies suggested that green infrastructure practices are both environmentally and economically beneficial and, in most cases, result in cost savings of 15–80% relative to conventional gray stormwater infrastructure (USEPA 2007).

Design of urban stormwater BMPs typically begins with consideration of rainfall recurrence intervals, which may be translated into design storm specifications or runoff depth. Practices are designed to achieve a level of service or performance associated with controlling a certain design storm, combination of design storms, and/or runoff depth to reduce flooding, stream erosion, and pollutant loading. The performance of stormwater practices is dictated primarily by precipitation intensity–duration–frequency (IDF) relationships, impervious surface area, and soils, along with life cycle maintenance (Claytor & Schueler 1996; Kadlec & Knight 1996; Roseen *et al*. 2009; Berndtsson 2010; Gallo *et al*. 2012; Hunt et al. 2012; Khan *et al*. 2012; Butcher 2021).

Stormwater design guidance has long recognized that land use change that increases impervious surface area leads to increased runoff and consequently greater capture requirements to achieve a specified level of service. Similarly, changes in precipitation characteristics should also result in changes to design criteria. Specifically, if the intensity of storms of a given frequency (e.g., a 25-year storm) or the frequency of storms of a given magnitude increases, then design specifications for BMPs based on historic climate will be inadequate to maintain intended performance based on retention volume and treatment contact times.

#### 3.1.2. Adaptability

As defined here, BMPs having a higher climate adaptation potential do not involve permanent or engineered structures, have either a shorter service lifespan or can be designed to respond and adapt to changing climatic conditions, or rely on more complex and redundant ecosystem processes that will adapt naturally to changing climatic conditions (e.g., vegetated systems designed to advance or recede in response to climate change). Urban BMPs can encompass a range of practices, from highly engineered concrete storage structures to plant-based systems such as bioretention cells. Permanent, engineered BMPs with long service lives are likely to have limited adaptation potential. For example, wet detention ponds require hard engineered structures with a pre-determined size and dedicated land area – and thus are generally difficult to adapt when climate changes. Proactive design of gray infrastructure to accommodate an expected change will increase costs but not necessarily guarantee adaptability if future conditions differ from expectations. Performance improvements can be made through advances such as real-time control systems, but there are limits to what can be achieved.

Adaptability of green infrastructure components such as bioretention can vary according to the type of vegetation that is used in the design. For example, trees increase evapotranspiration potential as their leaf area index and tree canopy increase with growth. Over time, they will mature, senesce, and need replacement. These factors should be considered, as well as selection of tree species, to have the widest possible range of growing conditions to encourage natural adaptation if rapid shifts in climate are anticipated. A bioretention cell involves an engineered design; however, the design is relatively easy to modify, the service lifespan is moderate, and pollutant removal is attained by a combination of physical filtration and uptake by plants (Davis *et al*. 2009).

In practice, various general stormwater adaptation strategies for urban areas have been proposed, mostly in locations outside of the US (Carmin *et al*. 2012). In the Netherlands, Gersonius *et al*. (2012) proposed a method for adapting gray stormwater infrastructure called the ‘mainstreaming method,’ which seeks to define the adaptive potential of infrastructure leading to phased approaches to address climate change. The method avoids single optimal strategies for adaptation, and instead suggests incorporating changes into projects at the time the project takes place (i.e., when aging infrastructure is replaced, urban revitalization, etc.). Other methods that have been proposed include an optimization scheme for BMPs (Karamouz *et al*. 2011), and adjusting design criteria in ways that result in oversizing for most of the design life (Mailhot & Duchesne 2010). Generally speaking, distributed stormwater approaches for adaptation such as green infrastructure, low impact development, and smart growth have been proposed as flexible, no-regrets strategies (Kessler 2011; Pyke *et al*. 2011; Butcher *et al*. 2013; Refsgaard *et al*. 2013).

### 3.2. Agricultural conservation practices

Agricultural conservation practices are typically implemented as a system at the landscape scale and are designed to improve crop growth and soil health as well as controlling pollutant loads. Here, we limit our discussion of resilience to individual practices for controlling pollutant loads from the agricultural landscape that occur as components of a larger system. Many agricultural conservation practices for controlling pollutant loads involve and interact strongly with ecosystem processes controlling crop growth and soil health. In addition, many agricultural conservation practices reduce or manage timing of flow and achieve pollutant removal through effects on ecosystem processes such as plant uptake and soil cover.

Key considerations for agriculture and climate change as reported in the National Climate Assessments (Melillo *et al*. 2014; USGCRP 2017) include:

- The *frost-free season* is projected to lengthen across much of the nation.
- The annual maximum number of *consecutive dry days* (less than 0.01 inches of rain) is projected to increase, especially in the western and southern parts of the nation. This trend will increase evaporation, decrease soil moisture levels, and add stress to limited water resources.
- Changes in *phenology* are expected. The timing of life cycle events in plants and animals, such as leaf-out, blooming, hibernation, and migration is likely to change. The recent trend of earlier springtime, milder winters, and longer growing seasons is expected to continue with significant impacts to agriculture and ecological systems.
- The US will experience changes in *plant and animal species composition and distribution* that are characteristic of specific areas.

Table 2 gives a summary of factors affecting the resilience of selected agricultural conservation practices. Additionally, more detailed information about the sensitivity, adaptability, and potential adaptive responses for different types of agricultural conservation practices, including reference citations, is available in the Supplementary material.

#### 3.2.1. Sensitivity

Many conservation practices have a dominant ecological and biotic component, such as plant uptake by cover crops and/or crop residue management, and interact strongly with soil biogeochemical and other ecosystem processes. Changes in climate potentially can affect the mobilization and transport of pollutants from upland areas to water bodies, as well as the structure and function of practices based on plant growth and other ecosystem processes. For instance, the filtration capacity of a forested riparian buffer depends on both the intensity of runoff entering the buffer, which is directly affected by climate, and the vigor of plant growth within the strip, which is indirectly affected by climate conditions. Thus, in series or parallel in a landscape, such practices can be sensitive to climate change in multiple and complex ways.

Agricultural conservation practice systems can also include storage components such as swales and constructed wetlands. These approaches are directly sensitive to the physical aspects of climate change, such as increases in precipitation intensity.

#### 3.2.2. Adaptability

Adaptability refers to the ease with which a practice can be modified, expanded, or otherwise altered to perform as intended over time as environmental conditions change. Many agricultural conservation practices, while sensitive to climate, are relatively adaptable because they are integrated parts of the seasonal regime of soil preparation, planting, and harvest. For example, the use of cover crops to provide erosion control can be evaluated annually and changes made as necessary as growth conditions change. This is not always the case, however, as certain changes will require significant investment in new equipment that is not readily accessible.

### 3.3. Forestry BMPs

BMPs for forestry typically focus on strategies to minimize human disturbance associated with forest harvest and road maintenance. Fire management and maintaining suitable habitat for cold water fisheries are other important areas of consideration for resilient, adaptable forestry management approaches. While a number of studies evaluate how forestry BMPs can be implemented at the landscape scale to mitigate effects of climate change (e.g., Swanston *et al*. 2016), relatively few explicitly address climate change effects on the performance of forestry BMPs.

Table 3 gives a summary of factors affecting resilience of selected forestry BMPs. Additionally, more detailed information about the sensitivity, adaptability, and potential adaptive responses for different types of forestry practices, including reference citations, is available in the Supplementary material.

#### 3.3.1. Sensitivity

Key factors affecting sensitivity to climate change include soil cover, organic matter content, and humidity distribution. An intact forest provides canopy and extensive organic litter cover that intercepts rainfall and protects the soil surface, a well-developed soil structure with dense networks of roots that help anchor soil, and secondary porosity associated with macropores and stump holes that encourages subsurface rather than overland flow. A harvest operation removes much of the cover and often compacts the soil, promoting greater overland flow and potential soil erosion. Removing cover can also alter soil moisture and increase temperature, resulting in changes in rates of decomposition and nutrient cycling. Likewise, management practices (such as Mechanical Vegetation Management and Post-Harvest BMPs) that influence soil cover will have a direct effect on ecosystem processes.

Soil organic matter (OM) is another important factor affecting BMP performance. Forest soils are generally 1–5% organic matter by weight and usually have higher OM than agricultural soils (Osman 2013). Soil OM performs a variety of physical, chemical, and biological functions including aggregation, soil reaction and ion exchange, nutrient cycling, and supplying food and energy to soil biota (Osman 2013).

Finally, humidity distribution and climate brittleness of a forest ecosystem can affect recycling of minerals, nutrients, and organic material and the ability of forests to recover from anthropogenic or natural disturbances (Savory & Butterfield 1999).

Forest ecosystems are vulnerable to various shifts in climate and extreme weather events. Changes in temperature, precipitation, and weather can influence risk of fires, disease, and pests, and change species composition and system structure. Tree mortality can alter evaporation, transpiration, and hydrologic processes including runoff and streamflow (Adams *et al*. 2012).

Die-off that is directly or indirectly caused by changes in precipitation (e.g., drought, pests) can result in regional-scale loss of overstory trees, which can change ecosystem type, properties, and land surface for years to decades (Breshears *et al*. 2005). Larger scale die-off events and ecosystem structure changes are predicted to occur in some areas under future warming conditions (Breshears *et al*. 2005).

#### 3.3.2. Adaptability

Forestry BMPs that rely on the re-establishment of mature riparian canopy, e.g., in streamside management zones, can take many decades to implement. This limitation can significantly reduce the adaptability of these practices. For example, a study of management strategies to maintain a temperature regime suitable for healthy salmonid populations in the South Fork Nooksack River (WA) found that substantial resilience to a warming climate could be provided by a mature riparian canopy; however, fully restoring a mature canopy would require decades of growth (Butcher *et al*. 2016).

In contrast, over shorter time horizons, forests can be vulnerable to climate-related impacts from pests, disease, fire, and wind. The combination of short time horizons for climate vulnerability and longer time horizons for recovery can render forest BMPs less easily adaptable.

BMPs that have high climate adaptation potential are generally those that minimize use of engineered structures, have a shorter service lifespan, and/or rely on more complex and redundant ecosystem processes. For example, forest roads and associated water management components such as culverts have low adaptation potential because they are engineered structures that are difficult to modify once built. A riparian buffer is a natural system that can be adapted to an evolving climate; however, the service lifespan can be extremely long, so flexibility to adjust to an altered climate is limited. In contrast, forest harvesting practices have a shorter planning horizon and can be modified in response to changing conditions.

## 4. RESEARCH NEEDS

In the course of this review, several topics were identified where additional study is needed. While not comprehensive, we note the following.

Studies directly assessing the effects of climate change (observed and projected) on practice performance are relatively limited. While basic principles about system response to changes in climatic drivers can be broadly applied, representative studies to inform local-scale adaptation planning are needed. Studies in underrepresented regions and watershed settings are particularly important to support adaptation planning in these areas.

Monitoring data are essential for documenting and understanding the long-term performance of practices in different regional and hydroclimatic settings. Such information can also assist localities and planners with justifying cost investments of practices, identifying adaptive management needs, and informing future decisions regarding siting and selection of new practices. Monitoring over a wide range of conditions is also key to calibrating hydrological and water quality models, particularly process-based models, which can subsequently be used to extrapolate potential changes in water quality and practices beyond current conditions. Existing, traditional monitoring, and especially long-term continuous monitoring programs, should be supported. Monitoring should also be expanded, where appropriate, to include targeted efforts to characterize practice performance across variations in weather and climate.

Much of the available literature on practice responses to climate change uses simulation models driven by climate projections. Simulation models are useful for understanding how practices might perform under an altered climate but are limited by the internal formulations of the models that may omit important processes. Evaluation of model simulations against field measurements, and improvements in our ability to simulate watershed hydrologic, water quality, and practice responses to climate change can advance our understanding of practice resilience on a broader scale. This is particularly important for practices that involve complex interactions between physical effects (e.g., increased flow) and biological effects (e.g., temperature-based changes in plant health and soil microbial processing rates). Water quality simulation models incorporate these effects in different ways (or not at all). Studies are needed to assess how well specific models can incorporate biological feedbacks on practice performance under altered climate regimes.

## 5. CONCLUSION

Faced with a tangible risk of climate change impacts, there is interest in strategies for increasing the resilience of water quality practices to a range of potential future conditions and events (Hoffman *et al*. 2014). In this review, we summarize the scientific literature addressing climate change effects on water quality practices commonly used to control runoff and pollutant loading from urban stormwater, agriculture, and forestry. This information is used to make qualitative inferences about the resilience of different water quality practices based on two criteria: sensitivity and flexibility/adaptability. Practices less sensitive to changes in climatic conditions will be more likely to function as intended as climate changes. More flexible/adaptable practices that can be revised or phased in over time provide a hedge against future risk.

For simplicity, we focus on individual types of water quality practices. Most often, however, practices are implemented with the goal of achieving a specified cumulative level of performance at a larger neighborhood to watershed scale, often described in the context of an overarching management plan or system of practices. Key to this is a systems (e.g., neighborhood or watershed based) approach, coupled with risk-based, adaptive planning (e.g., traditional adaptive management (Williams 2011), robust decision making (Groves & Lempert 2007)). Resilient practices, however, also are important components of such approaches. Ideally, practices should be designed to be flexible to changing needs at least cost and highest effectiveness.

Many factors must be considered in selecting practices to meet specific management goals including performance, cost, physical setting and related constraints, proximity to pollutant sources, and maintenance needs. Moreover, climate change effects on practices will also interact strongly with local changes in population, land use, water management infrastructure, and other factors. Changes in water availability and price may also have indirect effects on land management practices, such as irrigation, or result in tradeoffs between urban and agricultural water use. Climate change can also affect whether certain crops are appropriate in an area, while market forces may encourage either expansion or abandonment of farmland. Accordingly, it is important to evaluate adaptation strategies (including practices and other policy options) in a watershed-wide context against a range of possible land management and climate futures.

This review is intended to help communities incorporate consideration of resilience to climate change when planning to meet their water quality goals. Results are qualitative and do not contain specific, numeric guidance for adaptation planning. Rather, our goal is to benchmark and provide a foundation for evaluating potential climate change effects on water quality practices in different land use settings, thus helping planners and decision makers incorporate the potential implications of climate change on the design of individual practices and practice systems. Faced with the challenge of climate change, small, incrementally better decisions to increase the resilience of practices will, over time, yield tangible benefits. If pollutant loads increase under future climate, more resilient practices that perform as intended under altered conditions will minimize the need for potentially costly investments in structural modifications or additional practices. We hope, at a minimum, this review will help communities ask the right questions about current and future investments in water quality protection in the context of climate risk.

## Supplementary Material

SI

## Figures and Tables

**Table 1 |:** Summary of factors affecting resilience of selected urban BMPs

BMP Class	Climate Change Sensitivity	Adaptation Strategies
Biofiltration	• Performance of biofiltration practices is decreased by short runoff contact time, channelization, large storm events, frozen ground, short grass heights/sparse vegetative cover, and high runoff velocities and discharge rates • Changes in precipitation intensity could lead to concentration of sheet flow and increased transport of sediment and other contaminants	• Use flow diversion structures to bypass intense events, and/or increase size of pretreatment/energy dissipation structures • Alter vegetation species for drought and/or moisture tolerances • Provide supplemental irrigation during extreme drought periods • Modify maintenance and media replacement frequencies based on changes in decay rates, humidity distribution
Bioretention	• Changes in precipitation may affect retention time reducing effectiveness • Changes in soil moisture could affect infiltration capacity and plant uptake, while higher temperatures may amplify microbial activity in the soil media • Expansion potential may be limited in dense urban areas	• Alter vegetation species for drought and/or moisture tolerances • Provide supplemental irrigation during extreme drought periods • Adjust organic matter content of soil media • Modify maintenance and media replacement frequencies based on changes in decay rates, humidity distribution
Infiltration systems	• Changes in seasonal water table could affect infiltration capacity and increase risk of groundwater contamination • Rainfall fluctuations could affect design standards and make some existing facilities obsolete	• Incorporate flow diversion structures to bypass intense events, and/or increase size of pretreatment/energy dissipation structures • Where feasible, excavate to increase treatment volumes • Provide flow equalization storage at inlet • For vegetated systems, replant with species better adapted to climate
Green roofs	• Changes in temperature and precipitation may alter the species composition best suited for green roofs in a particular ecoregion • Greater precipitation volume and intensity may increase export of sediment/growth media and limit flow attenuation benefits	• Replant and adjust species composition to changing climate conditions • Incorporate downstream storage practices (e.g., cisterns) that help mitigate extreme event overflows and provide supplemental irrigation of green roof • Increase media organic matter content to improve moisture retention
Wet detention ponds	• Redesign of wet pond treatment volume and live storage in response to increased precipitation volume and intensity is likely to be difficult	• Retrofit outlet control structures and increase storage for new design storms • Supplement water during drought periods to maintain permanent pools
Dry detention ponds	• Redesign of pond treatment volume and live storage in response to increased precipitation volume and intensity is likely to be difficult	• Retrofit outlet control structures and increase storage for new design storms
Permeable pavement	• Treatment is controlled by infiltration rate; higher intensity storms may limit effectiveness	• If solids load increases, use pretreatment or divert flows from high load areas • Increase frequency of maintenance to reduce clogging
Grassed waterways	• More intense rainfall could increase concentrated flow erosion • Extended growing seasons could benefit functional processes, while significantly warmer temperatures could reduce soil cover and thus the overall effectiveness of the practice	• Retrofit to increase flow width and freeboard heights. • Modify outlet design to accommodate larger storms • Alter vegetation species composition to adapt to changing climate • Adjust mowing/grazing management as needed
Riparian buffers	• Extended growing seasons could improve filtration, while significantly higher temperatures could alter species composition and/or reduce soil cover/OM • More intense rainfall may promote concentrated flow through the buffer	• Increase up-gradient erosion control practices • Extend buffer widths, where feasible • Adjust species composition to adapt to altered climate
Constructed wetland	• Climate change may change suitability of native wetland plants and promote invasive or non-native species affect treatment capacity • Extended growing seasons could increase performance of constructed wetlands, but higher evaporation rates could alter desired water balance	• Incorporate flow diversion structures to bypass intense events, and/or increase size of pretreatment/energy dissipation structures • Provide flow equalization storage at inlet • Adjust plant species to match water balance changes • During extreme droughts, provide supplemental water to maintain pool

*Note*: See Supplementary material for references.

**Table 2 |:** Summary of factors affecting resilience of selected agricultural conservation practices

Conservation Practice	Climate Change Sensitivity	Adaptation Strategies
Conservation Till/No-till	• Increased temperature and sun exposure could more quickly dry residue, increasing wind transport, while more intense precipitation could increase loss via surface flow • A reduction in the climate ‘brittleness scale’ could hinder the ability of the residue to break down biologically	• Adjust timing of planting and residue termination dates to adjust for shifts in humidity and temperature • Increase crop stubble height to trap more snow • Produce more soil cover and organic matter via higher-residue crops and varieties and/or adjusting seeding rates and row spacing
Cover crops	• Warmer fall may increase establishment in cold areas • If rainfall is reduced, cover crops could begin competing for moisture and decrease the main crop’s moisture access • Increases in temperature could extend growing season for the main crop and reduce the effectiveness of cover crops • Increased decomposition rates could affect nutrient availability from cover crops	• Use cover crop species and varieties that are better adapted to the seasonal shifts in humidity and temperature • Adjust method and timing of cover crop planting and termination • Incorporate more biomass yielding species • Increase diversity of cover crop mixes • Incorporate more roll/crimp termination methods to preserve soil moisture
Perennial cropping	• Optimal species/variety for particular ecoregion could change over long term • Changes in precipitation intensity could lead to concentration of sheet flow via rill erosion, causing increased transport of sediment and other contaminants as well as reduced infiltration	• Replant with varieties better adapted to regional climate changes • Shorten slope lengths through use of terraces and keyline patterning • (Drier growing season) Install or improve irrigation as needed • Change ground cover management to increase water retention • Expand on-farm biodiversity to help mitigate pest/disease pressures
Nutrient Management Plans	• Unpredictable weather patterns could disrupt nutrient application schedules, and increased rainfall intensity would strengthen the likelihood of nutrient runoff	• Adjust application rates, sources, timing, and placement • Avoid manure application during periods when intense rainfall is likely • Increase frequency of soil and crop testing to improve efficiency • Incorporate conservation techniques (subsurface injection, no-till and residue management, etc.) that improve nutrient use efficiency
Controlled drainage	• Higher winter temperatures could increase denitrification and improve performance • Changes in hydrology and groundwater levels could alter optimal geographic/ecoregion placement of practice, as well as infrastructure sizing and design • Higher water tables may increase the release of soluble phosphorus from mineral soils	• Adjust water control structure elevations and timing of elevation shifts • Place practice in areas that are expected to have high water tables
Contour farming	• Increased precipitation intensity could exceed the ability of contours to control runoff and strengthen the potential for concentrated flow erosion	• Increase (wetter) or decrease (drier) row grade • Adjust ridge height, row spacing, and/or plant spacing within the row • Incorporate keyline patterning techniques to better distribute moisture • Expand use of residue/tillage management and no-till practices • Shorten slope lengths through use of diversions, terraces, etc. • Modify stable outlets to accommodate larger design storms
Grassed waterways	• Increased precipitation could increase concentrated flow erosion and cause the need to alter discharge capacity of grassed waterways • Extended growing seasons could benefit functional processes, while significantly warmer temperatures could reduce soil cover and thus the overall effectiveness of the practice	• Retrofit existing practices to increase flow width and freeboard heights • Modify outlet design to accommodate larger storms • Alter vegetation species composition (more wet or drought-tolerant species) • Adjust mowing/grazing management as needed to adapt
Riparian buffers	• Extended growing seasons could improve filtration, while significantly higher temperatures could alter species composition and/or reduce soil cover/OM • More intense rainfall may promote concentrated flow through the buffer	• Increase up-gradient erosion control practices • Extend buffer widths, where feasible • Adjust species composition to adapt to altered climate
Constructed wetland	• Climate change may change suitability of native wetland plants and promote invasive or non-native species affecting treatment capacity • Extended growing seasons could increase performance of constructed wetlands, but higher evaporation rates could alter desired water balance	• Incorporate flow diversion structures to bypass intense events, and/or increase size of pretreatment/energy dissipation structures • Provide flow equalization storage at inlet • Adjust plant species to match water balance changes • During extreme droughts, provide supplemental water

*Note*: See Supplementary material for references.

**Table 3 |:** Summary of factors affecting resilience of selected forestry BMPs

BMP Class	Climate Change Sensitivity	Adaptation Strategies
Wildland fire control and suppression	• Increased frequency and severity of drought will necessitate more severe suppression techniques, making it more difficult to minimize adverse effects • Drier conditions and unstable soil will increase the amount of soil disturbed and ground cover lost, further increasing erosion and runoff from suppression activities	• Increase public awareness about how wildfires may change with climate change as fire suppression efforts become more costly with warming climate • Remove active fuels from sites to control fires before they occur • Maintain natural fire regimes to reduce long-term intensities of wildfires • Manage fuel buildup by thinning or prescribed fires
Use of prescribed fire	• Increased frequency and severity of drought may limit the applicability of controlled burns as forests become drier, especially with increasing intensity and unpredictability of winds • Invasive species and pest infestations may increase difficulty in selectively administering controlled burns	• Promote use of controlled fires in areas where severe wildfires are expected • Consider use of mechanical thinning in forests with very high fuel loads and extreme drought
Road location and design	• Increased risk of road-related landslides and soil erosion due to severe and frequent precipitation, storm events	• Modify culvert size to reduce risk of flood damage • Avoid construction of roads near unstable soils to minimize risks of slope failure from precipitation and snowmelt
Stream crossings	• Changes in timing and volume of peak flows may damage infrastructure, pose threats to aquatic life, and impact potable water where stream crossings occur if they are not designed appropriately • Existing crossings may not be adequate if high flow events increase	• Increase culvert size below roads to reduce risk of flood damage to existing stream crossings and downstream resources • Evaluate established crossings to assess present suitability • Design stream crossings to be compatible with geomorphology of streams
Landing area management	• Soil is extensively disturbed at log landings, leading to erosion and runoff that is amplified by increased precipitation and storm events	• Scatter logging slash over landings and skid trails to stabilize and reduce erosion after operations • Consider placing landings a significant distance from streams likely to be affected by extreme precipitation events
Yarding operation	• Erosion may increase due to increasing frequency of heavy precipitation • Increased precipitation may also increase the hazard of slope failure in forested areas where ground-based operations are placed • Erosion may increase in select areas with permafrost melting	• Establish operational sites on stable soils • Consider precipitation and storm potential before establishing skidding and yarding infrastructure
Erosion prevention and control	• Mitigating the effects of mechanical vegetation treatment post-operation will likely increase in difficulty as changing precipitation and storm patterns increase erosion, runoff, soil instability, and slope failure	• Perform low-impact harvesting • Adjust harvest schedules to focus on winter harvesting • Consider partial harvests • Switch to pre-operation erosion prevention rather than post-operation control
Harvest unit planning and design	• Increased runoff and flooding after harvesting in response to increased heavy precipitation	• In cold areas use winter harvest to mitigate impacts of wet soils on harvesting • Reduce large-scale clearcutting • Promote natural regeneration • Increase rotation periods in coppices
Selective cutting	• Increased climatic variability, heavy precipitation and permafrost melt may lead to soil instability • Forests may require increased frequency and intensity of selective cutting due to increased prevalence of insect infestation and disease	• Modify harvest schedule to remove stands that are vulnerable to disturbance • Use persistent wood products to mitigate carbon losses when harvested • Adjust harvest schedules to winter-focused harvesting
Streamside management zones (SMZs)	• Heavy precipitation could increase flow velocity and decrease efficiency of buffer filtering and promote gullies in buffers • Increased tree mortality changes the width, density, and composition of buffers	• Increase buffer width and density where possible to enhance the ability of the buffer to absorb nutrients and filter sediment • Use Effective Function Width tool to assess and maintain effectiveness of stream buffers

*Note:* See Supplementary material for references.

## Data Availability

All relevant data are included in the paper or its Supplementary Information.

## HIGHLIGHTS

- Many water quality practices function via physical, chemical, and biological processes sensitive to weather and climate.
- Climate change presents a risk to practice-based investments in water quality protection; more resilient practices can help reduce this risk.
- Practice resilience can be characterized in terms of sensitivity to changes in weather and climate and ability to modify the practice over time.
- This review is intended to help communities and water resource managers consider climate resilience when adopting practices to meet water quality goals.
