# Supplementary material for: A review of climate change effects on practices for mitigating water quality impacts
Source: J Water Clim Chang. Author manuscript; Available in PMC 2023 Mar 22. (PMC9797054; doi:10.2166/wcc.2022.363)
Supplement: SI [file NIHMS1800634-supplement-SI.docx]

**ONLINE SUPPLEMENT**

A REVIEW OF CLIMATE CHANGE EFFECTS ON PRACTICES FOR MITIGATING WATER QUALITY IMPACTS

**Table of Contents:**

Section 1. Urban and Agricultural Practices…………………………………………………………1

Section 2. Forestry Practices……………………………………………………………………………..35

**Section 1: Urban and Agricultural Practices**

**Section 1.1: References - Urban and Agricultural**

Adeuya, R., N. Utt, J. Frankenberger, L. Bowling, E. Kladivko, S. Brouder, and B. Carter. 2012. Impacts of drainage water management on subsurface drain flow, nitrate concentration, and nitrate loads in Indiana. Journal of Soil and Water Conservation, 67: 474–484.

Alfredo, K., F. Montalto, and A. Goldstein. 2010. Observed and modeled performances of prototype greenroof test plots subjected to simulated low- and high-intensity precipitations in a laboratory experiment. Journal of Hydrologic Engineering, 15(6), 444–457

Alonso-Ayouso, M., M. Quemada, M. Vanclooster, M. Ruiz-Ramos, A. Rodriguez, and J.L. Gabriel. 2018. Assessing cover crop management under actual and climate change conditions. Science of the Total Environment, 621: 1330-1341.

Arisz, H., and B.C. Burrell. 2006. Urban drainage infrastructure planning and design considering climate change. In EIC Climate Change Technology, 2006 IEEE (pp. 1-9). doi: 10.1109/EICCCC.2006.277251.

Barber, M.E., S.G. King, D.R. Yonge, and W.E. Hathhorn. 2003. Ecology ditch: a best management practice for stormwater runoff mitigation. Journal of Hydrologic Engineering, 8(3): 111–122.

Berndtsson, J.C. 2010. Green roof performance towards management of runoff water quantity and quality: A review. Ecological Engineering, 36: 351-360.

Bosch, N.A., M.A. Evans, D. Scavia D., and J.D. Allan. 2014. Interacting effects of climate change and agricultural BMPs on nutrient runoff entering Lake Erie. Journal of Great Lakes Research, 40(3): 581-589.

Butcher, J., A. Parker, S. Sarkar, S. Job, M. Faizullabhoy, P. Cada, J. Wyss, R. Srinivasan, P. Tuppad, D. Debjani, A. Donigian, J. Imhoff, J. Kittle, B. Bicknell, P. Hummel, P. Duda, T. Johnson, C. Weaver, M. Warren, and D. Nover. 2013. Watershed Modeling to Assess the Sensitivity of Streamflow, Nutrient, and Sediment Loads to Potential Climate Change and Urban Development in 20 U.S. Watersheds. EPA/600/R-12/058F. National Center for Environmental Assessment, Office of Research and Development, U.S. Environmental Protection Agency, Washington, DC. Final, Sept. 30, 2013. http://cfpub.epa.gov/ncea/global/recordisplay.cfm?deid=256912.

California Stormwater Quality Association (CASQA). 2003. New Development and Redevelopment BMP Handbook. Menlo Park, CA.

Carmin, J., N. Nadkarni, C. Rhie. 2012. Progress and Challenges in Urban Climate Adaptation Planning: Results of a Global Survey. MIT Press, Cambridge, MA.

CASQA. 2003. Stormwater Best Management Practice Handbook, Municipal. California Stormwater Quality Association. https://www.casqa.org/sites/default/files/BMPHandbooks/BMP_Municipal_Complete.pdf.

CEAP: USDA Conservation Effects Assessment Project. https://www.nrcs.usda.gov/wps/portal/nrcs/main/national/technical/nra/ceap/.

Center for Watershed Protection. (n.d.). Stormwater Manager's Resource Center BMP Fact Sheets. Retrieved from www.stormwatercenter.net

Chiang, A.C., I. Chaubey, N.M. Hong, Y.P. Lin, and T. Huang. 2012. Implementation of BMP strategies for adaptation to climate change and land use change in a pasture-dominated watershed. International Journal of Environmental Research and Health, 9: 3654-3684.

Christianson, L.E., A. Bhandari, and M.J. Helmers. 2012. A practice-oriented review of woodchip bioreactors for subsurface agricultural drainage. Applied Engineering in Agriculture, 28(6): 861-874.

Clary, J., J. Jones, M. Leisenring, P. Hobson, and E. Strecker. 2017. International Stormwater BMP Database, 2016 Summary Statistics. Water Environment & Reuse Foundation, Alexandria, VA.

Claytor, R.A and Schueler, T.R. 1996. Design of Stormwater Filtering Systems. Chesapeake Research Consortium, Inc. Solomons, MD.

Cousino, L.A., R.H. Becker, and K.A. Zmijewski. 2015. Modeling the effects of climate change on water, sediment, and nutrient yields from the Maumee River watershed. Journal of Hydrology: Regional Studies, 4(B): 762-775.

Culligan, P.J. 2018. Green infrastructure and urban sustainability: Recent advances and future challenges. Proceedings, IBPC2018, Healthy, Intelligent and Resilient Buildings and Urban Environments. Syracuse, NY Sept. 23-26, 2018.

CWP. 2013. Stormwater Management Guidebook. Prepared for: District Department of the Environment, District of Columbia, by Center for Watershed Protection, Ellicott City, MD

Dakhlalla, A.O., and P.B. Parajuli. 2016. Evaluation of the best management practices at the watershed scale to attenuate peak streamflow under climate change scenarios. Water Resources Management, 30(3): 963-982.

D'Ambrosio, J.L.; Ward, A.; and J.D. Witter. 2013. A decade of benefits: Two-stage agricultural ditches in the Midwest Region of the United States. American Water Resources Association Annual Meeting. St. Louis, MO.

Delgado, J.A., P.M. Groffman, M.A. Nearing, T. Goddard, D. Reicosky, R. Lai, N.R. Kitchen, C.W. Rice, D. Towery, and P. Saton. 2011. Conservation practices to mitigate and adapt to climate change. Journal of Soil and Water Conservation, 66(4): 118A-128A.

Denault, C., R. G. Millar, and B.J. Lence. 2006. Assessment of possible impacts of climate change in an urban catchment. Journal of the American Water Resources Association, 42(3): 685-697.

Dorioz, J.M., D. Want, J. Poulenard, and D. Trévisan. 2006. The effect of grass buffer strips on phosphorus dynamics – A critical review and synthesis as a basis for application in agricultural landscapes in France. Agricultural Ecosystems & Environment, 117: 4-21.

Forsee, W.J. and S. Ahmad. 2011. Evaluating urban storm-water infrastructure design in response to projected climate change. Journal of Hydrologic Engineering, 16: 865-873.

Fuhrer, J. 2003. Agroecosystem responses to combinations of elevated CO2, ozone, and global climate change. Agriculture, Ecosystems and Environment, 97: 1–20.

Gallo, C., A. Moore, and J. Wywrot. 2012. Comparing the adaptability of infiltration based BMPs to various U.S. regions. Landscape and Urban Planning, 106(4): 326-335.

Garbrecht, J.D., M.A. Nearing, F.D. Shields Jr., M.D. Tomer, E.J. Sadler, J.V. Bonta, and C. Baffaut. 2014. Impact of weather and climate scenarios on conservation assessment outcomes. Journal of Soil and Water Conservation, 69(5): 374-392.

Gautam, S., E.G. Montagu, S. Kumar, J.V. Bonta, and R. Lal. 2015. Agricultural Policy Environmental eXtender model simulation of climate change impacts on runoff from a small no-till watershed. Journal of Soil and Water Conservation, 70: 101-109.

Gersonius, B., F. Nasruddin, R. Ashley, A. Jeuken, A. Pathirana, and C. Zevenbergen. 2012. Climate change uncertainty: Building flexibility into water and flood risk infrastructure. Climatic Change, 116(2): 411-423.

Gill, S.E., J.F. Handley, A.R. Ennos, and S. Pauleit. 2007. Adapting cities for climate change: The role of the green infrastructure. Built Environment, 33(1): 115-133. doi: 10.2146/benv.33.1.115.

Gilliam, J.W.; D.L. Osmond; and R.O. Evans. 1997. Selected Agricultural Best Management Practices to Control Nitrogen in the Neuse River Basin. North Carolina Agricultural Research Service Technical Bulletin 311. North Carolina State University, Raleigh, NC.

Gülbaz, S., and C.M. Kazezyılmaz-Alhan. 2017. Experimental investigation on hydrologic performance of LID with rainfall-watershed-bioretention system. Journal of Hydrologic Engineering, 22(1), D4016003.

Ha, M., and M. Wu. 2017. Land management strategies fo rimproving water quality in biomass production under changing climate. Environmental Research Letters 12, 034015.

Hamlet, A.F., and D.P. Lettenmaier. 2007. Effects of 20th century warming and climate variability on flood risk in the western US. Water Resources Research, 43:W06427.

Hatfield, J.L., and J.H. Prueger. 2004. Impacts of changing precipitation on water quality. Journal of Soil and Water Conservation, 59(1): 51-58.

Hodaj, A.; L.C. Bowling; C. Raj; and I. Chaubey. 2016. Evaluation of the Two Stage Ditch as a Best Management Practice. A Doctoral Dissertation Submitted to the Faculty of Purdue University by Andi Hodaj. Purdue University, West Lafayette, Indiana.

Horsley Witten Group, Inc. 2015. Assessment of Climate Change Impacts on Stormwater BMPs and Recommended BMP Design Considerations in Coastal Communities. A report prepared for the Massachusetts Office of Coastal Zone Management, Boston, MA.

Hoss, F., J. Fischbach, and E. Molina-Perez. 2016. Effectiveness of best management practices for stormwater treatment as a function of runoff volume. Journal of Water Resources Planning and Management, 142(11)

Hunt , W. F., Davis, A.P., and R.G. Traver. 2012. Meeting hydrologic and water quality goals through targeted bioretention design. Journal of Environmental Engineering, 138(6): 698-707.

Iowa State University Cooperative Extension. 2011. Woodchip Bioreactors for Nitrate in Agricultural Drainage. Extension and Outreach Factsheet. Ames, Iowa.

IPCC. 2014. Summary for Policymakers. In: Climate Change 2014: Impacts, Adaptation, and Vulnerability. Part A: Global and Sectoral Aspects. Contribution of Working Group II to the Fifth Assessment Report of the Intergovernmental Panel on Climate Change [Field, C.B., V.R. Barros, D.J. Dokken, K.J. Mach, M.D. Mastrandrea, T.E. Bilir, M. Chatterjee, K.L. Ebi, Y.O. Estrada, R.C. Genova, B. Girma, E.S. Kissel, A.N. Levy, S. MacCracken, P.R. Mastrandrea, and L.L.White (eds.)]. Cambridge University Press, Cambridge, United Kingdom and New York, NY, USA, pp. 1-32.

Jaynes, D.B., and T.M. Isenhart. 2014. Reconnecting tile drainage to riparian buffer hydrology for enhanced nitrate removal. Journal of Environmental Quality, 43: 631-638.

Kadlec, R. H, and R.L. Knight. 1996. Treatment Wetlands. Lewis Publishers, Boca Raton, FL.

Karamouz, M., A. Hosseinpour, and S. Nazif. 2011. Improvement of urban drainage system performance under climate change impact: Case study. Journal of Hydrological Engineering, 16: 395-412.

Kessler, R. 2011. Stormwater strategies: Cities prepare against aging infrastructure for climate change. Environmental Health Perspectives, 119(12): A514-A519.

Khan, U. T., C. Valeo, A. Chu, and B. van Duin. 2012. Bioretention cell efficacy in cold climates: Part 1 — hydrologic performance. Canadian Journal of Civil Engineering, 39(11): 1210-1221.

King County. 2014. Evaluation of Potential Climate Change Impacts on Stormwater Management. Prepared by Jeff Burkey, Science and Technical Support Section, Water and Land Resources Division. Seattle, WA.

Kleerekoper, L., M. van Esch, and T.B. Salcedo. 2012. How to make a city climate-proof: addressing the urban heat island effect. Resources, Conservation, and Recycling, 64: 30-38. doi: 10.1016/j.resconrec.2011.06.004

Kovacic, D.; David, M.; Gentry, L.; Starks, K.; and R. Cooke. 2000. Effectiveness of constructed wetlands in reducing nitrogen and phosphorus export from agricultural tile drainage. Journal of Environmental Quality, 29(4): 1262-1274.

Kristvik, E., G.H. Kleiven, J. Lohne, and T.M. Muthanna. 2018. Assessing robustness of raingardens under climate change using SDSM and temporal downscaling. Water Science & Technology, 77: 1640-1650

Lal, R., J.A. Delgado, P.M. Groffman, N. Millar, C. Dell C., and A. Rotz. 2011. Management to mitigate and adapt to climate change. Journal of Soil and Water Conservation, 66(4): 276-285.

Lee, K.H., T.M. Isenhart, R.C. Schultz, and S.K. Mickelson. 2000. Multispecies riparian buffers trap sediment and nutrients during rainfall simulations. Journal of Environmental Quality, 29: 1200-1205.

Lee, S., A.M. Sadeghi, L.-Y. Yeo, W. McCarty, and W.D. Hively. 2017. Assessing the impacts of future climate conditions on the effectiveness of winter cover crops in reducing nitrate loads into Chesapeake Bay watersheds using the SWAT model. Transactions of the ASABE, 60: 1939-1955.

Liu, Y., W. Yang, C. Qin, and A. Zhu. 2016. A review and discussion on modeling and assessing agricultural best management practices under global climate change. Journal of Sustainable Development, 9(1): 245-255.

Mahl, U.H., J.L. Tank, S.S. Roley, and R.T. Davis. 2015. Two-stage ditch floodplains enhance N-removal capacity and reduce turbidity and dissolved P in agricultural streams. Journal of the American Water Resources Association, 51: 923–940.

Mailhot, A., and S. Duchesne. 2010. Design criteria of urban drainage infrastructures under climate change. Journal of Water Resources Planning and Management, 136: 201-208.

MDA. 2017. Agricultural BMP Handbook for Minnesota . Minnesota Dept. of Agriculture, St. Paul, MN.

Melillo, J.M., T.C. Richmond, and G.W. Yohe, eds. 2014: Climate Change Impacts in the United States: The Third National Climate Assessment. U.S. Global Change Research Program, 841 pp. doi:10.7930/J0Z31WJ2.

Mellander, P.E., P. Jordan, M. Shore, S.R. Melland, and G. Shortle. 2015. Flow paths and phosphorus transfer pathways in two agricultural streams with contrasting flow controls. Hydrological Processes, 29: 3504-3518.

Nangia, V., D.J. Mulla, and P.H. Gowda. 2010. Precipitation changes impact stream discharge, nitrate–nitrogen load more than agricultural management changes. Journal of Environmental Quality, 39: 2063-2071.

Nearing, M.A. 2001. Potential changes in rainfall erosivity in the U.S. with climate change during the 21st century. Journal of Soil and Water Conservation, 56: 229-232.

Nearing, M.A., F.F. Pruski, and M.R. O’Neal. 2004. Expected climate change impacts on soil erosion rates: a review. Journal of Soil and Water Conservation, 59(1): 43-50.

Nearing, M.A., V. Jetten, C. Baffaut, O. Cerdan, A. Couturier, M. Hernandez, Y. Le Bissonnais, M.H. Nichols, J.P. Nunes, C.S. Renschler, and V. Souchère. 2005. Modeling response of soil erosion and runoff to changes in precipitation and cover. Catena, 61(2): 131-154.

Newcomer, M. E., J.J. Gurdak, L.S. Sklar, and L. Nanus. 2014. Urban recharge beneath low impact development and effects of climate variability and change. Water Resources Research, 50: 1-16.

North Carolina Department of Environment and Natural Resources Division of Water Quality. 2007. Stormwater Best Management Practices Manual. Raleigh, NC.

NRCS FOTGs. Field Office Technical Guides. https://www.nrcs.usda.gov/wps/portal/nrcs/main/national/technical/fotg/

O’Neal, M.R., M.A. Nearing, R.C. Vining, J. Southworth J., and R.A. Pfiefer. 2005. Climate change impacts on soil erosion in Midwest United States with changes in crop management. Catena, 61: 165-184.

Penn State University. 1996. Nutrient Management in Conservation Tillage Systems. Conservation Tillage Series Number 4. Penn State Cooperative Extension, University Park, PA.

Penn State University. 2006. Cover Crops for Conservation Tillage Systems. Conservation Tillage Series Number 5. Penn State Cooperative Extension, University Park, PA.

Philadelphia Water. 2018. Stormwater Management Guidance Manual, version 3.1

Pielke, R.A. Sr. 2013. Mesoscale Meteorological Modeling, 3rd ed. Academic Press/Elsevier

Porter, J.H., M.L. Parry, and T.R. Carter. 1991. The potential impacts of climate change on agricultural insect pests. Agricultural and Forest Meteorology, 57: 221-240.

Pruski, F.F., and M.A. Nearing. 2002. Climate-induced changes in erosion during the 21st century for eight U.S. locations. Water Resources Research, 38(12).

Pyke, C., M. P. Warren, T. Johnson, J. LaGro Jr., J. Scharfenberg, P. Grothd, R. Freede, W. Schroeere, and E. Main. 2011. Assessment of low impact development for managing stormwater with changing precipitation due to climate change. Landscape and Urban Planning, 103(2): 166-173.

Refsgaard, J. C., K. Arnbjerg-Nielsen, M. Drews, K. Halsnæs, E. Jeppesen, H. Madsen, A. Markandya, J.E. Olesen, J. R. Porter, and J.H. Christensen. 2013. The role of uncertainty in climate change adaptation strategies – A Danish water management example. Mitigation and Adaptation Strategies for Global Change, 18(3): 337–359.

Roley, S.S., J.L. Tank, N.A. Griffiths, R.O. Hall Jr., and R.T. Davis. 2014. The influence of floodplain restoration on whole-stream metabolism in an agricultural stream: Insights from a 5-yr continuous data set. Freshwater Science, 33(4): 1043-1059

Roseen, R.M., T.P. Ballestero, J.J. Houle, P. Avellaneda, J. Briggs, G. Fowler, and R. Wildey. 2009. Seasonal performance variations for storm-water management systems in cold climate conditions. Journal of Environmental Engineering, 135: 128-137.

Rosenzweig, C., and D. Hillel. 2000. Soils and global climate change: Challenges and opportunities. Soil Science, 165(1): 47-56.

Rossman, L.A. 2014. National Stormwater Calculator User’s Guide—Version 1.1. EPA/600/R-13/085b. US Environmental Protection Agency, Water Supply and Water Resources Division, Cincinnati, OH.

Rounsevell, M.D.A., S.P. Evans, and P. Bullock. 1999. Climate change and agricultural soils: impacts and adaptation. Climate Change, 43: 683-709.

Savory, A., and J. Butterfield. 1999. Holistic Management: A New Framework for Decision Making. Island Press. ISBN 978-1-55963-488-5.

Schmidt, M.L., S. Sarkar, J.B. Butcher, T.E. Johnson, and S.H. Julius. 2019. Agricultural best management practice sensitivity to changing air temperature and precipitation. Transactions of the ASABE, 62(4): 1021-1033.

Semadeni-Davies, A. 2012. Implications of climate and urban development on the design of sustainable urban drainage systems (SUDS). Journal of Water and Climate Change, 3(4): 239-256.

Semadeni-Davies, A., C. Hernebring, G. Svensson, and L.-G. Gustafsson. 2008a. The impacts of climate change and urbanisation on drainage in Helsingborg, Sweden: Combined sewer system. Journal of Hydrology, 350(1-2): 100-113.

Semadeni-Davies, A., C. Hernebring, G. Svensson, and L.-G. Gustafsson. 2008b. The impacts of climate change and urbanisation on drainage in Helsingborg, Sweden: Suburban stormwater. Journal of Hydrology, 350(1-2): 114-125.

Sharma, A. K., L. Vezzaro, H. Birch, K. Arnbjerg-Nielsen, and P.S. Mikkelsen. 2011. Effect of climate change on stormwater characteristics and treatment efficiencies of stormwater retention ponds. 12th International Conference on Urban Drainage. Porto Alegre/Brazil.

Sohn, W., J.-Y. Kim, M.-H. Li, and R. Brown. 2019. The influence of climate on the effectiveness of low impact development: A systematic review. Journal of Environmental Management, 236: 365-379.

Soil and Water Conservation Society. 2006. Planning for Extremes: A Report from a Soil and Water Conservation Society Workshop Held in Milwaukee, Wisconsin, November 1-3, 2006.

State of Washington Department of Ecology. 2014. Stormwater Management Manual. Lacey, WA.

Taha, H. 1997. Urban climates and heat islands: albedo, evapotranspiration, and anthropogenic heat. Energy and Buildings, 25: 99-103.

Tilman, D., K.G. Cassman, P.A. Matson, R. Naylor, and S. Polasky. 2000. Agricultural sustainability and intensive production practices. Nature, 418: 671-677.

Tomer, M.D., E.J. Sadler, R.E. Lizotte, R.B. Bryant, T.L. Potter, M.T. Moore, T.L. Veith, C. Baffaut, M.A. Locke, and M. R. Walbridge. 2014. A decade of conservation effects assessment research by the USDA Agricultural Research Service: progress overview and future outlook. Journal of Soil and Water Conservation, 69(5): 365-373.

Tugel, A.J., A.M. Lewandowski, and D. Happe-von Arb, eds. 2000. Soil Biology Primer. Ankeny, IA: Soil and Water Conservation Society.

U.S. Department of Agriculture (USDA) Natural Resources Conservation Service (NRCS). (n.d.). Conservation Effects Assessment Project (CEAP) Cropland National Assessment. Retrieved from https://www.nrcs.usda.gov/wps/portal/nrcs/detail/national/technical/nra/ceap/na/?cid=nrcs143_014144

U.S. Department of Agriculture (USDA) Natural Resources Conservation Service (NRCS). (n.d.). Conservation Effects Assessment Project (CEAP) Grazing Lands National Assessment. Retrieved from https://www.nrcs.usda.gov/wps/portal/nrcs/detail/national/technical/nra/ceap/na/?cid=nrcs143_014159

U.S. Department of Agriculture (USDA) Natural Resources Conservation Service (NRCS). (n.d.). National Conservation Practice Standards. Retrieved from https://www.nrcs.usda.gov/wps/portal/nrcs/detailfull/national/technical/cp/ncps/?cid=nrcs143_026849

U.S. Environmental Protection Agency. 2007. Reducing Stormwater Costs through Low Impact Development (LID) Strategies and Practices. EPA 841-F-07-006. Nonpoint Source Control Branch, Washington, DC.

Van Liew, M.W., S. Feng, and T.B. Pathak. 2012. Climate change impacts on streamflow, water quality, and best management practices for the Shell and Logan Creek watersheds in Nebraska. International Journal of Agricultural and Biological Engineering, 5(1): 13-34.

Vanuytrecht, E., C. Van Mechelen, K. Van Meerbeek, P. Willems, M. Hermy, and D. Raes. 2014. Runoff and vegetation stress of green roofs under different climate change scenarios. Landscape and Urban Planning, 122: 68-77.

Wallace, C.W., D.C. Flanagan, and B.A. Engel. 2017. Quantifying the effects of conservation practice implementation on predicted runoff and chemical losses under climate change. Agricultural Water Management, 186: 51-65.

Walsh, C.J., A. Roy, J. Feminella, P.D. Cottingham, P.F. Groffman, and R.P. Morgan II. 2005. The urban stream syndrome: Current knowledge and the search for a cure. Journal of the North American Benthological Society, 24(3):706−723

Wang, R., X. Zhang, and M.-H. Li. 2019. Predicting bioretention pollutant removal efficiency with design features: A data-driven approach. Journal of Environmental Management, 242: 403-414.

Washington State University Extension Service and Puget Sound Partnership. 2012. Low Impact Development Technical Guidance Manual for Puget Sound.

Waters, D., W. E. Watt, J. Marsalek, and B.C. Anderson. 2003. Adaptation of a storm drainage system to accommodate increased rainfall resulting from climate change. Journal of Environmental Planning and Management, 46(5): 755-770.

Woznicki S.A., and A.P. Nejadhashemi. 2012. Sensitivity analysis of best management practices under climate change scenarios. Journal of the American Water Resources Association, 48(1): 90-112.

Woznicki, S.A., A.P. Nejadhashemi, and C.M. Smith. 2011. Assessing best management practice implementation strategies under climate change scenarios. Transactions of the ASABE, 54(1): 171-190.

Woznicki, S.A., and A.P. Nejadhashemi. 2014. Assessing uncertainty in best management practice effectiveness under future climate scenarios. Hydrological Processes, 28: 2550-2566.

Zhang, X.C., W.Z. Liu, Z. Li, and J. Chen. 2011. Trend and uncertainty analysis of simulated climate change impacts with multiple GCMs and emission scenarios. Agricultural and Forest Meteorology, 151: 1297-1304.

**Section 2: Forestry Practices**

**Section 2.1: References – Forestry**

Adams, H.D., C.H. Luce, D.D. Breshears, C.D. Allen, M. Weiler, V.C. Hale, A.M.S. Smith, and T.E. Huxman. 2012. Ecohydrological consequences of drought- and infestation-triggered tree die-off: Insights and hypotheses. Ecohydrology 5:145–159.

Akay, A.E., and J. Sessions. 2005. Applying the decision support system, TRACER, to forest road design. Western Journal of Applied Forestry 20(3):184–191.

AFC. 2007. Alabama’s Best Management Practices for Forestry. Alabama Forestry Commission, Montgomery, AL.

Allen, C.D., A.K. Macalady, H. Chenchouni, D. Bachelet, N. McDowell, M. Vennetier, T. Kitzberger, A. Rigling, D.D. Breshears, E.H. Hogg, P. Gonzalez, R. Fensham, Z. Zhang, J. Castro, N. Demidova, J.-H. Lim, G. Allard, S.W. Running, A. Semerci, and N. Cobb. 2010. A global overview of drought and heat-induced tree mortality reveals emerging climate change risks for forests. Forest Ecology and Management 259:660–684.

Bartkowiak, S.M., L.J. Samuelson, M.A. McGuire, and R.O. Teskey. 2015. Fertilization increases sensitivity of canopy stomatal conductance and transpiration to throughfall reduction in an 8-year-old loblolly pine plantation. Forest Ecology and Management 354:87–96.

Bentz, B.J., J. Regniere, C.J. Fettig, E.M. Hansen, J.L. Hayes, J.A. Hicke, R.G. Kelsey, J.F. Negron, and S.J. Seybold. 2010. Climate change and bark beetles of the western United States and Canada: Direct and indirect effects. Bioscience 60(8):602–613.

Bessie, W.C., and E.A. Johnson. 1995. The relative importance of fuels and weather on fire behavior in subalpine forests. Ecology 76(3):747–762.

Black, A. 2004. Wildland Fire Use: The ‘‘Other’’ Treatment Option. Environmental Consequences Fact Sheet 6, Fuels Planning: Science Synthesis and Integration. Res. Note RMRS-RN-23-6-WWW. Department of Agriculture, Forest Service, Rocky Mountain Research Station, Fort Collins, CO, U.S.

Boisvenue, C., and S.W. Running. 2010. Simulations show decreasing carbon stocks and potential for carbon emissions in Rocky Mountain forests over the next century. Ecological Applications 20(5):1302–1319.

Bonan, G.B. 2008. Forests and climate change: Forcings, feedbacks, and the climate benefits of forests. Science 320:1444–1449.

Borja, M.E.L. 2014. Climate change and forest natural regeneration in Mediterranean mountain areas. Forest Research 3(2), 1000e108.

Breshears, D.D., N.S. Cobb, P.M. Rich, K.P. Price, C.D. Allen, R.G. Balice, W.H. Romme, J.H. Kastens, M.L. Floyd, J. Belnap, J.J. Anderson, O.B. Myers, and C.W. Meyer. 2005. Regional vegetation die-off in response to global-change-type drought. PNAS 102(42):15144–15148.

Carey, H., and M. Schumann. 2003. Modifying Wildfire Behavior – The Effectiveness of Fuel Treatments: The Status of Our Knowledge. National Community Forestry Center Southwest Region Working Paper 2.

CASQA. 2003. Stormwater Best Management Practice Handbook, Municipal. California Stormwater Quality Association. https://www.casqa.org/sites/default/files/BMPHandbooks/BMP_Municipal_Complete.pdf.

CEC. n.d. Ecological Regions of North America. Commission for Environmental Cooperation.

Chan, S.S., D.J. Larson, K.G. Maas-Hebner, W.H. Emmingham, S.R. Johnston, and D.A. Mikowski. 2006. Overstory and understory development in thinned and underplanted Oregon Coast Range Douglas-fir stands. Canadian Journal of Forest Research 36(10):2696–2711.

Chizinski, C.J., B. Vondracek, C.R. Blinn, R.M. Newman, D.M. Atuke, K. Fredricks, N.A. Hemstad, E. Merten, and N. Schlesser. 2010. The influence of partial timber harvesting in riparian buffers on macroinvertebrate and fish communities in small streams in Minnesota, USA. Forest Ecology and Management 259(10):1946–1958.

Coutts, M.P., C.C.N. Nielsen, and B.C. Nicoll. 1999. The development of symmetry, rigidity, and anchorage in the structural root system of conifers. Plant and Soil 217:1–15.

Cristan, R., W. M. Aust, M.C. Bolding, S. M. Barrett, J.F. Munsell, and E. Schilling. 2016. Effectiveness of forestry best management practices in the United States: Literature review. Forest Ecology and Management 360:133–151.

Dale, V.H., L.A. Joyce, S. McNulty, R.P. Neilson, M.P. Ayres, M.D. Flannigan, P.J. Hanson, L.C. Irland, A.E. Lugo, C.J. Peterson, D. Simberloff, F.J. Swanson, B.J. Stocks, and B.M. Wotton. 2001. Climate change and forest disturbances. BioScience 51(9):723–734.

Doppelt, B., R. Hamilton, C.D. Williams, M. Koopman, and S. Vynne. 2009. Preparing for Climate Change in the Upper Willamette River Basin of Western Oregon: Co-Beneficial Planning for Communities and Ecosystems. U.S. Department of Agriculture, Climate Leadership Initiative, and National Center for Conservation Science and Policy.

Dore, S., T.E. Kolb, M. Montes-Helu, S.E. Eckert, B.W. Sullivan, B.A. Hungate, J.P. Kaye, S.C. Hart, G.W. Koch, and A. Finkral. 2010. Carbon and water fluxes from ponderosa pine forests disturbed by wildfire and thinning. Ecological Applications 20(3):663–683.

Dressing, S.A. 2003. National Management Measures for the Control of Nonpoint Pollution from Agriculture. EPA-841-B-03-004. Office of Water, U.S. Environmental Protection Agency.

Everham, E.M., III, and N.V.L. Brokaw. 1996. Forest damage and recovery from catastrophic wind. Botanical Review 62(2):113–185.

Ferrell, G.T. 1996. Chapter 45: The Influence of Insect Pests and Pathogens on Sierra Forests. In Sierra Nevada Ecosystem Project: Final Report to Congress, vol. II, Assessments and Scientific Basis for Management Options. Davis: University of California, Centers for Water and Wildland Resources.

Friedenberg, N.A., J.A. Powell, and M.P. Ayres. 2007. Synchrony’s double edge: Transient dynamics and the allee effect in stage structured populations. Ecology Letters 10:564–573.

Froberg, M., P.J. Hanson, D.E. Todd, and D. W. Johnson. 2008. Evaluations of effects of sustained decadal precipitation manipulations on soil carbon stocks. Biogeochemistry 89:151–161.

Fulton, S., and B. West. [2002.] AQUA-3: Forestry Impacts on Water Quality. Chapter AQUA-3 in Southern Forest Resource Assessment. USDA Forest Service Southern Research Station and Southern Region. https://www.srs.fs.usda.gov/sustain/draft/aqua3/aqua3.pdf.

Gara, R.I., D.R. Millegan, and K.E. Gibson. 1999. Integrated pest management of Ips pini (Col., Scolytidae) populations in south-eastern Montana. Journal of Applied Entomology 123:529–534.

Garnas, J.R., M.P. Ayres, A.M. Liebhold, and C. Evans. 2011. Subcontinental impacts of an invasive tree disease on forest structure and dynamics. Journal of Ecology 99:532–541.

Gillooly, J.F., E.L. Charnov, G.W. West, W.M. Savage, and J.H. Brown. 2002. Effects of size and temperature on developmental time. Nature 417:70–73.

Grace, J.M., III, and B.D. Clinton. 2007. Protecting soil and water in forest road management. Transactions of the American Society of Agricultural and Biological Engineers 50(5):1579-1584.

Grace, J.M., III. 2005. Forest operations and water quality in the south. Transactions of the American Society for Agricultural Engineers 48(2):871–880.

Grace, J.M., III, R.W. Skaggs, and G. M. Chescheir. 2006. Hydrologic and Water Quality Effects of Thinning Loblolly Pine. Transactions of the American Society of Agricultural and Biological Engineers 49(3):645–654.

Grodzki, W. 1997. Pityogenes chalcographus (Coleoptera, Scolytidae) – an indicator of man-made changes in Norway spruce stands. Biologia, Bratislava 52:217–220.

Gumus, S., H.H. Ascar, and D. Toksoy. 2008. Functional forest road network planning by consideration of environmental impact assessment for wood harvesting. Environmental Monitoring and Assessment 142:109–116.

Hanson, P.J., and J.F. Weltzin. 2000. Drought disturbance from climate change: Response of United States forests. The Science of the Total Environment 262:205–220.

Hanson, P.J., T.J. Tschaplinski, S.D. Wullschleger, D.E. Todd, Jr., and R. M Auge. 2007. The resilience of upland-oak forest canopy trees to chronic and acute precipitation manipulations. Proceedings of the 15th Central Hardwood Forest Conference.

Harpold, A.A. 2016. Diverging sensitivity of soil water stress to changing snowmelt timing in the Western U.S. Advances in Water Resources 92:116–129.

Holden, Z.A., C.H. Luce, M.A. Crimmins, and P. Morgan. 2012. Wildfire extent and severity correlated with annual streamflow distribution and timing in the Pacific Northwest, USA (1984-2005). Ecohydrology 5:677–684.

Hu, J., D.J.P. Moore, S.P. Burns, and R.K. Monson. 2010. Longer growing seasons lead to less carbon sequestration by a subalpine forest. Global Change Biology 16:771–783.

Iverson, L.R., A.M. Prasad, S. N. Matthews, and M. Peters. 2008. Estimating potential habitat for 134 eastern US tree species under six climate scenarios. Forest Ecology and Management 254:390–406.

Jactel, H., B.C. Nicoll, M. Branco, J.R. Gonzalez-Olabarria, W. Grodzki, B. Langstrom, F. Moreira, S. Netherer, C. Orazio, D. Piou, H. Santos, M.J. Schedlhaas, K. Tojic, and F. Vodde. 2009. The influence of forest stand management on biotic and abiotic risks of damage. Annals of Forest Science, Springer Verlag/EDP Sciences 66(7):701.

Johnson, D.W., D.E. Todd, Jr., and P.J. Hanson. 2008. Effects of throughfall manipulation on soil nutrient status: results of 12 years of sustained wet and dry treatments. Global Change Biology 14: 1661-1675.

Jones, J.A., and D.A. Post. 2004. Seasonal and successional streamflow response to forest cutting and regrowth in the northwest and eastern United States. Water Resources Research 40(5), W05203.

Jones, J.A., G.L. Achterman, L.A. Augustine, I.F. Creed, P.F. Ffolliott. L. MacDonald, and B.C. Wemple. 2009. Hydrologic effects of a changing forested landscape – challenges for the hydrological sciences. Hydrological Processes 23:2699–2704.

Kolstrom, M., M. Lindner, T. Vilen, M. Maroschek, R. Seidl, M.J. Lexer, S. Netherer, A. Kremer, S. Delzon, A. Barbati, M. Marchetti, and P. Corona. 2011. Reviewing the Science and Implementation of Climate Change Adaptation Measures in European Forestry. Forests 2:961–982.

Leinenbach, P. 2016. Memorandum to Allan Henning, USEPA regarding shade loss and temperature increase resulting from the implementation of Option A and Option B of the proposed Oregon Forest Practices Rule for SSTB streams in sections of western Oregon. U.S. Environmental Protection Agency.

Lindner, M., M. Maroschek, S. Netherer, A. Kremer, A. Barbati, J. Garcia-Gonzalo, R. Seidl, S. Delzon, P. Corona, M. Kolstrom, M.J. Lexer, and M. Marchetti. 2010. Climate change impacts, adaptive capacity, and vulnerability of European forest ecosystems. Forest Ecology and Management 259:698–709.

Linohss, A.C., A. Cameron, H. Hall, S. Blair, and T. Ankersen. 2012. Large woody material: Science, policy, and best management practices for Florida streams. Florida Scientist 75:157–175.

Liu, X., X. Zhang, and M. Zhang. 2008. Major factors influencing the efficacy of vegetated buffers on sediment trapping: A review and analysis. Journal of Environmental Quality 37:1667–1674.

Liu, Y., J. Stanturf, and S. Goodrick. 2010. Trends in global wildfire potential in a changing climate. Forest Ecology and Management 259:685–697.

Marin County. 2007. Memorandum of Understanding Among the Marin Municipal Water District, County of Marin, Marin County Open Space District, California Department of Parks and Recreation, National Park Service, and Marin County Resource Conservation District for Woody Debris Management in Riparian Areas of the Lagunitas Creek Watershed. Best Management Practices for Woody Debris in Riparian Areas of Salmon Bearing Streams of the Lagunitas Creek Watershed Final: February 1, 2007

Marion, D.A., G. Sun, P.V. Caldwell, C.F. Miniat, Y. Ouyang, D.M. Amatya, B.D. Clinton, P.A. Conrads, S.G. Laird, Z. Dai, J.A. Clingenpeel, T. Liu, E.A. Roehl Jr., J.A.M. Meyers, and C. Trettin. 2013. Managing forest water quantity and quality under climate change. Chapter 9 in Climate Change Adaptation and Mitigation Management Options Guide for Natural Resource Managers in Southern Forest Ecosystems 249–306.

McDowell, N., W.T. Pockman, C.D. Allen, D.D. Breshears, N. Cobb, T. Kolb, J. Plaut, J. Sperry, A. West, D.G. Williams, and E. A. Yepez. 2008. Mechanisms of plant survival and mortality during drought: Why do some plants survive while others succumb to drought? New Phytologist 178:719–739.

Meixner, T. 2004. Wildfire Impacts on Water Quality. Southwest Hydrology 24–25.

Melillo, J.M., T.C. Richmond, and G.W. Yohe, eds. 2014. Climate Change Impacts in the United States: The Third National Climate Assessment. U.S. Global Change Research Program, doi:10.7930/J0Z31WJ2.

Mote, P.W., E.A. Parson, A.F. Hamlet, W.S. Keeton, D. Lettenmaier, N. Mantua, E.L. Miles, D. W. Peterson, D.L. Peterson, R. Slaughter, and A.K. Snover. 2003. Preparing for climatic change: The water, salmon, and forests of the Pacific Northwest. Climatic Change 61:45–88.

NASF. 2017a. Protecting Water Quality through State Forestry Best Management Practices. National Association of State Foresters. http://stateforesters.org/sites/default/files/issues-and-policies-documents-of-interest/Protecting_Water_Quality_through_State_Forestry_BMPs_FINAL.pdf.

NASF. 2017b. Map of Forestry Best Management Practices by State. National Association of State Foresters. Accessed July 2017. http://stateforesters.org/action-issues-and-policy/state-forestry-BMPs-map.

NCFA. 2017. Forest Management Basics. North Carolina Forestry Association. Accessed May 11, 2017. https://www.ncforestry.org/teachers/forest-management-basics/.

Nearing, M.A., V. Jetten, C. Baffaut, O. Cerdan, A. Couturier, M. Hernandez, Y. Le Bissonnais, M.H. Nichols, J.P. Nunes, C.S. Renschler, and V. Souchère. 2005. Modeling response of soil erosion and runoff to changes in precipitation and cover. Catena, 61(2):131–154.

Neary, D.G., G.G. Ice, and C.R. Jackson. 2009. Linkages between forest soils and water quality and quantity. Forest Ecology and Management 258(10):2269–2281.

Newton, M. and J.A. Norgren. 1977. Silvicultural Chemicals and Protection of Water Quality. EPA 910/9-77-036. U.S. Environmental Protection Agency, Seattle, WA.

Obrist, D., C. Pearson, J. Webster, T. Kane, C.-J. Lin, G.R. Aiken, and C.N. Alpers. 2016. Terrestrial mercury in the western United States: Spatial distribution defined by land cover and plant productivity. Science of the Total Environment, 508: 522-535.

O’Gorman, P.A., and T. Schneider. 2009. The physical basis for increases in precipitation extremes in simulations of 21st-century climate change. PNAS 106(35):14773–14777.

Ochterski, J. 2004a. Best Management Practices After the Timber Harvest. Cornell Cooperative Extension. Accessed May 12, 2017. http://www2.dnr.cornell.edu/ext/bmp/contents/postharvest/post_intro.htm.

Ochterski, J. 2004b. Best Management Practices During a Timber Harvest. Cornell Cooperative Extension. Accessed June 8, 2017. http://www2.dnr.cornell.edu/ext/bmp/contents/during/dur_intro.htm

Ogden, A.E., and J.L. Innes. 2008. Climate change adaptation and regional forest planning in southern Yukon, Canada. Mitigation and Adaptation Strategies for Global Change 13(8):833-861.

Osborne, L.L., and D.A. Kovacic. 1993. Riparian vegetated buffer strips in water-quality restoration and stream management. Freshwater Biology 29:243–258.

Osman, K.T. 2013. Organic matter of forest soils. Pp. 63-96 in Forest Soils: Properties and Management. Springer International Publishing. doi: 10.1007/978-3-319-02541-4_4.

Paashaus, E., R. Briggs, and N. Ringler. 2004. Partial cutting impacts on macroinvertebrates in ephemeral streams in southern NY. In: Proceedings: Forestry across Borders: New England Society of American Foresters 84th Winter Meeting. Northeastern Research Station.

Palmer, M.A., D.P. Lettenmaier, N.L. Poff, S.L. Postel, B. Richter, and R. Warner. 2009. Climate change and river ecosystems: Protection and adaptation options. Environmental Management 44:1053–1068.

Paradis, A., J. Elkinton, K. Hayhoe, and J. Buonaccorsi. 2008. Role of winter temperature and climate change on the survival and future range expansion of the hemlock woolly adelgid (Adelges tsugae) in eastern North America. Mitigation and Adaptation Strategies for Global Change 13:541–554.

Parker, T.J., K.M. Clancy, and R.L. Mathiasen. 2006. Interactions among fire, insects, and pathogens in coniferous forests of the interior western United States and Canada. Agricultural and Forest Entomology 8:167–189.

Peltola, H., B. Gardiner, S. Kellomaki, T. Kolstrom, R. Lassig, J. Moore, C. Quine, and J.-C. Ruel. 2000. Wind and other abiotic risks to forests. Forest Ecology and Management 135:1–2.

Phillips, M.J., L.W. Swift, Jr., and C.R. Blinn. 2000. Best Management Practices for Riparian Areas, pp. 273–286 in: Riparian Management in Forests of the Continental Eastern United States, ed. E.S. Verry, J.W. Hornbeck, and C. Andrew. Lewis Publishers, CRC Press LLC, Boca Raton, FL.

Pinay, G., and H. Decamps. 1988. The role of riparian woods in regulating nitrogen fluxes between the alluvial aquifer and surface water: A conceptual model. Regulated Rivers: Research and Management 2:507–516.

Pryor, S.C., and R.J. Barthelmie. 2010. Climate change impacts on wind energy: A review. Renewable and Sustainable Energy Reviews 14:430–437.

Raffa, K.F., B.H. Aukema, B.J. Bentz, A.L. Carroll, J.A. Hicke, M.G. Turner, and W.H. Romme. 2008. Cross-scale drivers of natural disturbances prone to anthropogenic amplification: The dynamics of bark beetle eruptions. BioOne 58(6):501–517.

Rier, S.T., N.C. Tuchman, and R.G. Wetzel. 2005. Chemical changes to leaf litter from trees grown under elevated CO2 and the implications for microbial utilization in a stream ecosystem. Canadian Journal of Fisheries and Aquatic Sciences 62:185–194.

Rosenzweig, C., and D. Hillel. 2000. Soils and global climate change: Challenges and opportunities. Soil Science, 165(1):47–56.

Rothermel, R.C. 1983. How to Predict the Spread and Intensity of Forest and Range Fires. General Technical Report INT-143, U.S. Department of Agriculture, Ogden, UT.

Rustad, L., J. Campbell, J.S. Dukes, T. Huntington, K.F. Lambert, J. Mohan, and N. Rodenhouse. 2011. Changing Climate, Changing Forests: The Impacts of Climate Change of Forests of the Northeastern United States and Eastern Canada. General Technical Report NRS-99, U.S. Department of Agriculture, Northern Research Station, Newtown Square, PA.

Sabater, S., A. Butturini, J.-C. Clement, T. Burt, D. Dowrick, M. Hefting, V. Maitre, G. Pinay, C. Postolache, M. Rzepecki, and F. Sabater. 2003. Nitrogen removal by riparian buffers along a European climatic gradient: Patterns and factors of variation. Ecosystems 6:20–30.

Safranyik, L., A.L. Carroll, J. Regniere, D.W. Langor, W.G. Riel, T.L. Shore, B. Peter, B.J. Cooke, V.G. Nealis, and S.W. Taylor. 2010. Potential for range expansion of mountain pine beetle into the boreal forest of North America. BioOne 142(5):415–442.

Savory, A., and J. Butterfield. 1999. Holistic Management: A New Framework for Decision Making. Island Press. ISBN 978-1-55963-488-5.

Schönau, A.P.G., and J. Coetzee. 1989. Initial spacing, stand density and thinning in eucalypt plantations. Forest Ecology and Management 29(4):245–266.

Schowalter, T.D., W.H. Hargrove, and D. A. Crossley, Jr. 1986. Herbivory in forested ecosystems. Annual Review of Entomology 31:177–196.

Seavy, N.E., T. Gardali, G.H. Golet, F.T. Griggs, C.A. Howell, R. Kelsey, S.L. Small, J.H. Viers, and J.F. Weigand. 2009. Why climate change makes riparian restoration more important than ever: Recommendations for practice and research. Ecological Restoration 27(3):330–338.

Seidl, R., and W. Rammer. 2016. Climate change amplifies the interactions between wind and bark beetle disturbances in forest landscapes. Landscape Ecology doi: 10.1007/s10980-016-0396-4.

Six, D.L., M.V. Meer, T.H. DeLuca, and P. Kolb. 2002. Pine engraver (Ips pini) colonization of logging residues created using alternative slash management systems in western Montana. Western Journal of Applied Forestry 17(2): 96–100.

SDSU. 2003. Forestry Best Management Practices for South Dakota. South Dakota State University, College of Agriculture and Biological Sciences.

Spittlehouse, D.L., and R.B. Stewart. 2003. Adaptation to climate change in forest management. BC Journal of Ecosystems and Management 4(1):1–11.

Standing Forestry Committee (SFC) Ad Hoc Working Group III on Climate Change and Forestry. 2010. Climate Change and Forestry. Report to the Standing Forestry Committee, European Commission.

Stocks, B.J. 1987. Fire potential in the spruce budworm-damaged forests of Ontario. The Forestry Chronicle 63(1): 8–14.

Sturrock, R.N., S.J. Frankel, A.V. Brown, P.E. Hennon, J.T. Kliejunas, K.J. Lewis, J.J. Worrall, and A.J Woods. 2011. Climate change and forest diseases. Plant Pathology 60:133–149.

Swanston, C., M.K. Janowiak, L.A. Brandt, P.R. Butler, S.D. Handler, P.D. Shannon, A.D. Lewis, K. Hall, R.T. Fahey, L. Scott, and A. Kerber. 2016. Forest Adaptation Resources: Climate Change Tools and Approaches for Land Managers. General Technical Report NRS-87-2. U.S. Department of Agriculture, Newton Square, PA.

Swetnam, T.W., and A.M. Lynch. 1993. Regional-scale patterns of western spruce budworm outbreaks. Ecological Monographs 63(4):399–424.

Swetnam, T.W., and K.L. Betancourt. 1997. Mesoscale disturbance and ecological response to decadal climatic variability in the American Southwest. Journal of Climate 11:3128–3147.

Swift, L.W., Jr. 1985. Forest road design to minimize erosion in the Southern Appalachian. Pp. 141-151 in Proceedings of Forestry and Water Quality; A Mid-south Symposium, ed. B.G. Blackmon, Monticello, AR: University of Arkansas.

Switalski, T.A., J.A. Bissonette, T.A. DeLuca, C.H. Luce, and M.A. Madej. 2004. Benefits and impacts of road removal. Frontiers in Ecology 2(1):21–28.

Tchakarian, M.D., and R.N. Coulson. 2011. Chapter 15: Ecological Impacts of Southern Pine Beetle. Pp 223-234 in: Southern Pine Beetle II. General Technical Report SRS-140, ed. R.N. Coulson and K.D. Klepzig. U.S. Department of Agriculture Forest Service, Southern Research Stations.

Tran, J.K., T. Ylioja, R.F. Billings, J. Regniere, and M.P. Ayres. 2007. Impact of minimum winter temperatures on the population dynamics of Dendroctonus frontalis. Ecological Applications 17(3):882–899.

UAF. 2017. Keeping Forests Sustainable. University of Alaska Fairbanks. Spring 2017 Research Highlights, Fairbanks, AK.

UNH. 2009. New Hampshire Stream Crossing Guidelines. University of New Hampshire.

USDA. 2012. National Best Management Practices for Water Quality Management on National Forest System Lands. Volume 1: National Core BMP Technical Guide. FS-990a, U.S. Department of Agriculture.

USEPA. 2005. National Management Measures to Control Nonpoint Source Pollution from Forestry. United State Environmental Protection Agency. Office of Water, U.S. Environmental Protection Agency.

USEPA, and CEC. 2017. Ecological Regions of North America: Level I. U.S. Environmental Protection Agency and the Commission for Environmental Compliance.

USFS. 2011. Appendix F: United States Forest Service Requirements. U.S. Forest Service.

USFS. 2012. Future of America’s Forests and Rangelands: 2010 Resources Planning Act Assessment. General Technical Report WO-87. U.S. Department of Agriculture, U.S. Forest Service, Washington, DC.

USGCRP. 2009. Global Climate Change Impacts in the United States. T.R. Karl, J.M. Melillo, and T.C. Peterson, Eds. United States Global Change Research Program, Cambridge University Press, New York.

USGS. 1997. Effects of Climate Change on Southeastern Forests. USGS FS 093-97. U.S. Geological Survey.

VDOF. 2009. Virginia’s Forestry Best Management Practices for Water Quality: Field Guide. Virginia Department of Forestry.

Vose, J.M., C.R. Ford, S. Laseter, S. Dymond, G. Sun, M.B. Adams, S. Sebestyen, J. Campbell, C. Luce, D. Amatya, K. Elder, and T. Heartsill Scalley. 2012a. Can forest watershed management mitigate climate change effects on water resources? In Proceedings of a Workshop held during the XXV IUGG General Assembly, International Union of Geodesy and Geophysics, Melbourne, June–July 2011. International Association of Hydrological Sciences Publication 353. Oxfordshire, UK.

Vose, J.M., D.L. Peterson, and T. Patel-Weynand. 2012b. Effects of Climatic Variability and Change on Forest Ecosystems: A Comprehensive Science Synthesis for the U.S. Forest Sector. General Technical Report PNW-GTR-870. United Stated Department of Agriculture, Forest Service, Portland, Oregon.

Walsh, J., D. Wuebbles, K. Hayhoe, J. Kossin, K. Kunkel, G. Stephens, P. Thorne, R. Vose, M. Wehner, J. Willis, D. Anderson, S. Doney, R. Feely, P. Hennon, V. Kharin, T. Knutson, F. Landerer, T. Lenton, J. Kennedy, and R. Somerville. 2014. Ch. 2: Our Changing Climate. Climate Change Impacts in the United States: The Third National Climate Assessment.

Ward, E.J., J.-C. Domec, M.A. Laviner, T.R. Fox, G. Sun, S. McNulty, J. King, and A. Noormets. 2015. Fertilization intensifies drought stress: Water use and stomatal conductance of Pinus taeda in a midrotation fertilization and throughfall reduction experiment. Forest Ecology and Management 355:72–82.

Weatherspoon, C.P., and C.N. Skinner. 1995. An assessment of factors associated with damage to tree crowns from the 1987 wildfires in Northern California. Forest Science 41(3):430–451.

Weltzin, J.F., M.E. Loik, S. Schwinning, D.G. Williams, P.A. Fay, B.M. Haddad, J. Harte, T.E. Huxman, A.K. Knapp, G. Lin, W.T. Pocklman, M.R. Shaw, E.E. Small, M.D. Smith, S.D. Smith, D.T. Tissue, and J.C. Zak. 2003. Assessing the response of terrestrial ecosystems to potential changes in precipitation. BioOne 53(10):941–952.

Westerling, A.L., A. Gershunov, T.J. Brown, D.R. Cayan, and M.D. Dettinger. 2003. Climate and wildfire in the western United States. Journal of the American Meteorological Society 595–604.

Winchell, T.S., D.M. Barnard, R.K. Monson, S. P. Burns, and N.P. Molotch. 2016. Earlier snowmelt reduces atmospheric carbon uptake in midlatitude subalpine forests. Geophysical Research Letters 43:8160–8168.

Woodall, C.W., C.M. Oswalt, J.A. Westfall, C.H. Perry, M.D. Nelson, and A.O. Finley. 2009. An indicator of tree migration in forests of the eastern United States. Forest Ecology and Management 257:1434–1444.

Wu, Z., P. Dijkstra, G.W. Koch, J. Penuelas, and B.A. Hungate. 2011. Responses of terrestrial ecosystems to temperature and precipitation change: A meta-analysis of experimental manipulation. Global Change Biology 17:927–942.

Ylioja, T., D.H. Slone, and M.P. Ayres. 2005. Mismatch between herbivore behavior and demographics contributes to scale-dependence of host susceptibility in two pine species. Forest Science 51(6): 522–531.

Young-Robertson, J.M., W.R. Bolton, U.S. Bhatt, J. Cristobal, and R. Thoman. 2016. Deciduous trees are a large and overlooked sink for snowmelt water in the boreal forest. Scientific Reports 6, #29504.
